# Statins improve cardiac endothelial function to prevent heart failure with preserved ejection fraction through upregulating circRNA-RBCK1

**DOI:** 10.1038/s41467-024-47327-z

**Published:** 2024-04-05

**Authors:** Bin Li, Wen-Wu Bai, Tao Guo, Zhen-Yu Tang, Xue-Jiao Jing, Ti-Chao Shan, Sen Yin, Ying Li, Fu Wang, Mo-Li Zhu, Jun-Xiu Lu, Yong-Ping Bai, Bo Dong, Peng Li, Shuang-Xi Wang

**Affiliations:** 1 State Key Laboratory for Innovation and Transformation of Luobing Theory, the Key Laboratory of Cardiovascular Remodeling and Function Research, Chinese Ministry of Education, Chinese National Health Commission and Chinese Academy of Medical Sciences, Department of Cardiology, Qilu Hospital of Shandong University, Jinan, China; 2 Department of Cardiology, Central Hospital Affiliated to Shandong First Medical University, Jinan, Shandong China; 3 College of Pharmacy, Henan International Joint Laboratory of Cardiovascular Remodeling and Drug Intervention, Xinxiang Key Laboratory of Vascular Remodeling Intervention and Molecular Targeted Therapy Drug Development, Xinxiang Medical University, Xinxiang, Henan China; 4 Department of Geriatric Medicine, National Clinical Research Center for Geriatric Disorders, Xiangya Hospital of Central South University, Changsha, Hunan China; 5 Department of Cardiology, Shandong Provincial Hospital Affiliated to Shandong First Medical University, Jinan, Shandong China

**Keywords:** Heart failure, Mechanisms of disease, Non-coding RNAs

## Abstract

Heart failure with preserved ejection fraction (HFpEF) is associated with endothelial dysfunction. We have previously reported that statins prevent endothelial dysfunction through inhibition of microRNA-133a (miR-133a). This study is to investigate the effects and the underlying mechanisms of statins on HFpEF. Here, we show that statins upregulate the expression of a circular RNA (circRNA-RBCK1) which is co-transcripted with the ring-B-box-coiled-coil protein interacting with protein kinase C-1 (RBCK1) gene. Simultaneously, statins increase activator protein 2 alpha (AP-2α) transcriptional activity and the interaction between circRNA-RBCK1 and miR-133a. Furthermore, AP-2α directly interacts with RBCK1 gene promoter in endothelial cells. In vivo, lovastatin improves diastolic function in male mice under HFpEF, which is abolished by loss function of endothelial AP-2α or circRNA-RBCK1. This study suggests that statins upregulate the AP-2α/circRNA-RBCK1 signaling to suppress miR-133a in cardiac endothelial cells and prevent diastolic dysfunction in HFpEF.

## Abstract (web summary)

Endothelial dysfunction has been shown to occur in HFpEF and we know that statins can target endothelial dysfunction by inhibiting miR-133a. Here the authors show that statins improve diastolic dysfunction in HFpEF by increasing the levels of a circRNA which, in turns, binds to miR-133a modulating its downstream targets.

## Introduction

Heart failure with preserved ejection fraction (HFpEF) is currently the most prevalent form of heart failure in the world^1–3^. Although the clinical characteristics of HFpEF are somewhat heterogeneous, diastolic dysfunction is one of the most important features^4,5^. Endothelial dysfunction, characterized as endothelium-derived nitric oxide (NO) deficiency, is assessed as a deficient vasodilatory response to various stimuli^6,7^. Beyond vascular endothelial cells, endothelial cells in the heart warrant considerations for their roles in HFpEF^8,9^. Therefore, drugs targeting cardiac endothelial cells would be considerable to prevent HFpEF in clinical.

Statins increase NO bioavailability via tetrahydrobiopterin (BH4)-mediated endothelial NO synthase (eNOS) recoupling^10–12^, inhibit endomyocardial inflammation, and lower cardiomyocyte resting tension^13^. We have also reported that statins suppress miRNA-133a ectopic expression in vascular endothelial cells to prevent endothelial dysfunction through targeting GTP cyclohydrolase 1 (GTPCH1)^14^. Considering the importance of endothelial dysfunction in multiple cardiovascular diseases^15,16^, we thought that statins upregulated GTPCH1 gene expression to prevent cardiac endothelial dysfunction in HFpEF through miR-133a inhibition in cardiac endothelial cells.

Non-coding RNAs, such as circular RNA (circRNA), were firstly detected in virus as covalently closed looped RNAs that show complex tissue- and stage-specific expression within the eukaryotic transcriptome^17,18^. A number of circRNAs have been identified as functional molecules in regulating disease progression through circRNA-miRNA duplex, which enables circRNAs to serve as “miRNA sponge” and prevents miRNA-targeted degradation of mRNAs^19–21^. It promotes us to assume that statins-mediated miR-133a inhibition in cardiac endothelial cells is driven by circRNA as a sponge.

In this work, we hypothesized that statins prevent cardiac endothelial dysfunction to improve diastolic function through circRNA-mediated miR-133a suppression. Here, we reported that statins, such as lovastatin, activate transcriptional factor activator protein 2 alpha (AP-2α) to increase hsa_circRNA_102979 gene expression, which binds miR-133a to upregulate GTPCH1 gene expression in cardiac endothelial cells. In this way, statins improve diastolic dysfunction to prevent HFpEF.

## Results

### Statins upregulate circRNA-RBCK1 expression in endothelial cells

**Fig. 1:**
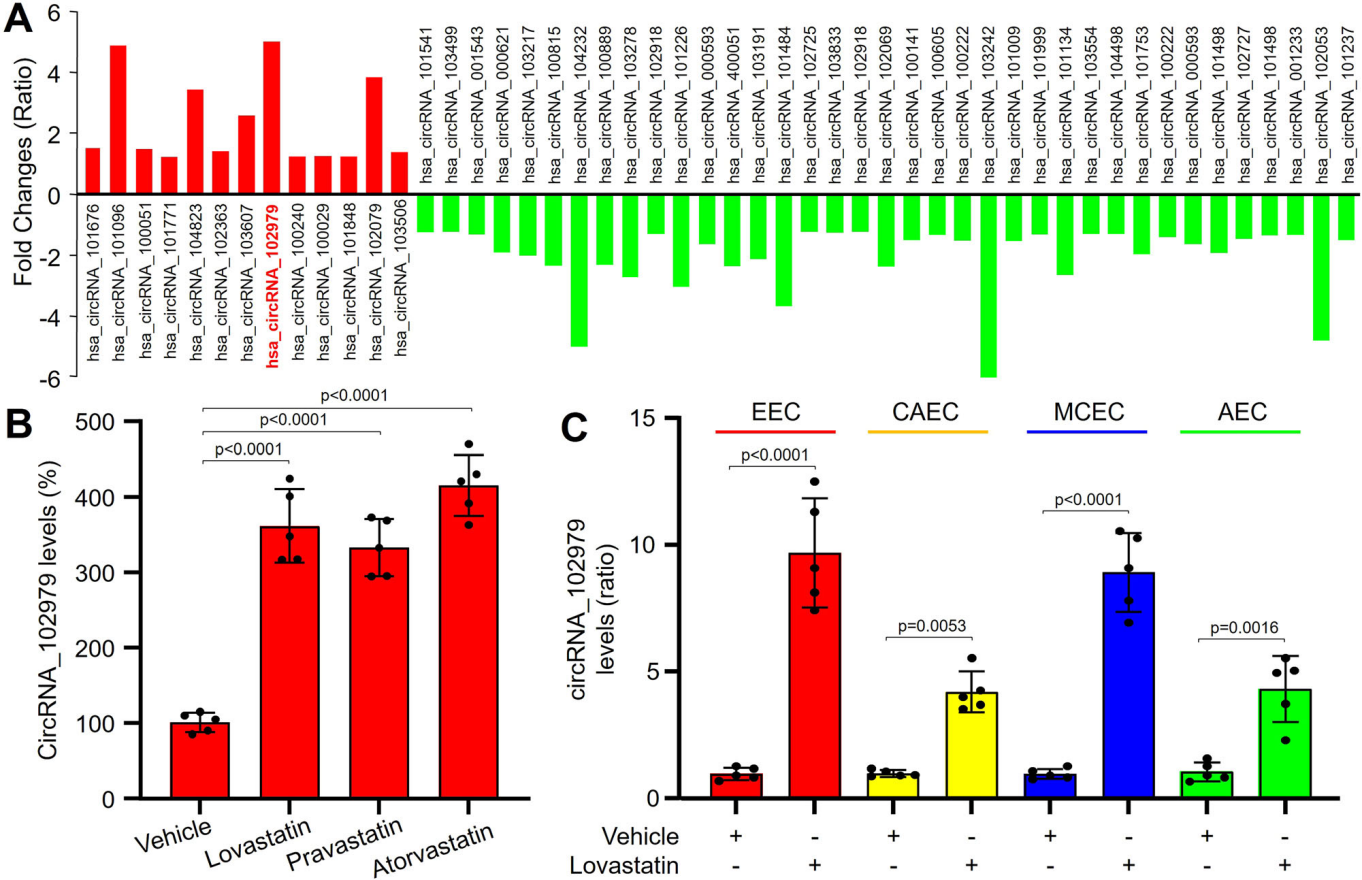
Statins increase circRNA-RBCK1 gene expression in endothelial cells. **A** Cultured human umbilical vein endothelial cells (HUVECs) were treated lovastatin (10 μM) for 24 h. Total RNAs were extracted and subjected to perform RNA sequencing analysis. 13 circRNAs were increased in red and 37 circRNAs were decreased in green. **B** Cultured HUVECs were treated with lovastatin (10 μM), pravastatin (20 μM), and atorvastatin (10 μM) for 24 h. **C** Primary human endocardium endothelial cells (EEC), human coronary arterial endothelial cells (CAEC), human myocardial capillary endothelial cells (MCEC), and human aortic endothelial cells (AEC) were incubated with lovastatin (10 μM) for 24 h. The levels of circRNA-RBCK1 were measured by quantitative PCR in (**B**) and (**C**). All experiments were repeated from five different donors per cell type. A one-way ANOVA followed by the Dunnett test was used to determine *P* value in (**B**). A two-sided unpaired Student’s *t* test was used to determine *P* value in (**C**). Data are presented as mean ± SD. Source data are provided as a Source Data file.

To test the hypothesis, we first examined the effects of lovastatin on the expressions of GTPCH1 mRNA and miR-133a in cardiac endothelial cells and vascular endothelial cells. Interestingly, statins upregulated GTPCH1 gene expression in cardiac endothelial cells, but the miR-133a expression was not dramatically suppressed in cardiac endothelial cells as vascular endothelial cells (Supplementary Fig. 1A, B). We also examined the effect of lovastatin on circRNA expressional profiles in human umbilical vein endothelial cells (HUVECs) by performing RNA sequencing analysis (Supplementary Data 1). As indicated in Fig. 1A, a total of 13 circRNAs were increased, while 37 circRNAs were decreased after lovastatin treatment. Among these circRNAs, hsa_circRNA_102979 (also called hsa_circ_0059151 and mm9_circ_003522) was increased the mostly by lovastatin. The augmentative effect of lovastatin on hsa_circRNA_102979 gene expression was further confirmed by other statins such as pravastatin and atorvastatin (Fig. 1B). Because hsa_circRNA_102979 is co-transcripted in chr20:398169-409233 with ring-B-box-coiled-coil protein interacting with protein kinase C-1 (RBCK1) gene^22^, we named hsa_circRNA_102979 as circRNA-RBCK1.

Endothelial cells in the heart include vascular endothelium, e.g., coronary arterial endothelial cells (CAECs), and cardiac endothelium including endocardium endothelial cells (EECs) and myocardial capillary endothelial cells (MCECs). Both EECs and MCECs communicate with subjacent cardiomyocytes directly to regulate cardiac function through endothelial-myocardial signaling^8,23^. Therefore, we compared the effects of statins on circRNA-RBCK1 expressions between vascular endothelium and cardiac endothelium. In Fig. 1C, lovastatin upregulated circRNA-RBCK1 in four types of endothelial cells. Surprisingly, the effects of lovastatin on circRNA-RBCK1 in cardiac endothelial cells were much stronger than in vascular endothelial cells.

### CircRNA-RBCK1 binds miR-133a in endothelial cells

To determine which miRNA is a target of circRNA-RBCK1, we performed a computational target-scan analysis to predict the miRNAs targeted by circRNA-RBCK1 using the CircNet database. Five miRNAs including miR-125a, miR-133a, miR-502, miR-637, and miR-1301 could potentially bind to the highly conserved target sites within circRNA-RBCK1 (Supplementary Fig. 2).

**Fig. 2:**
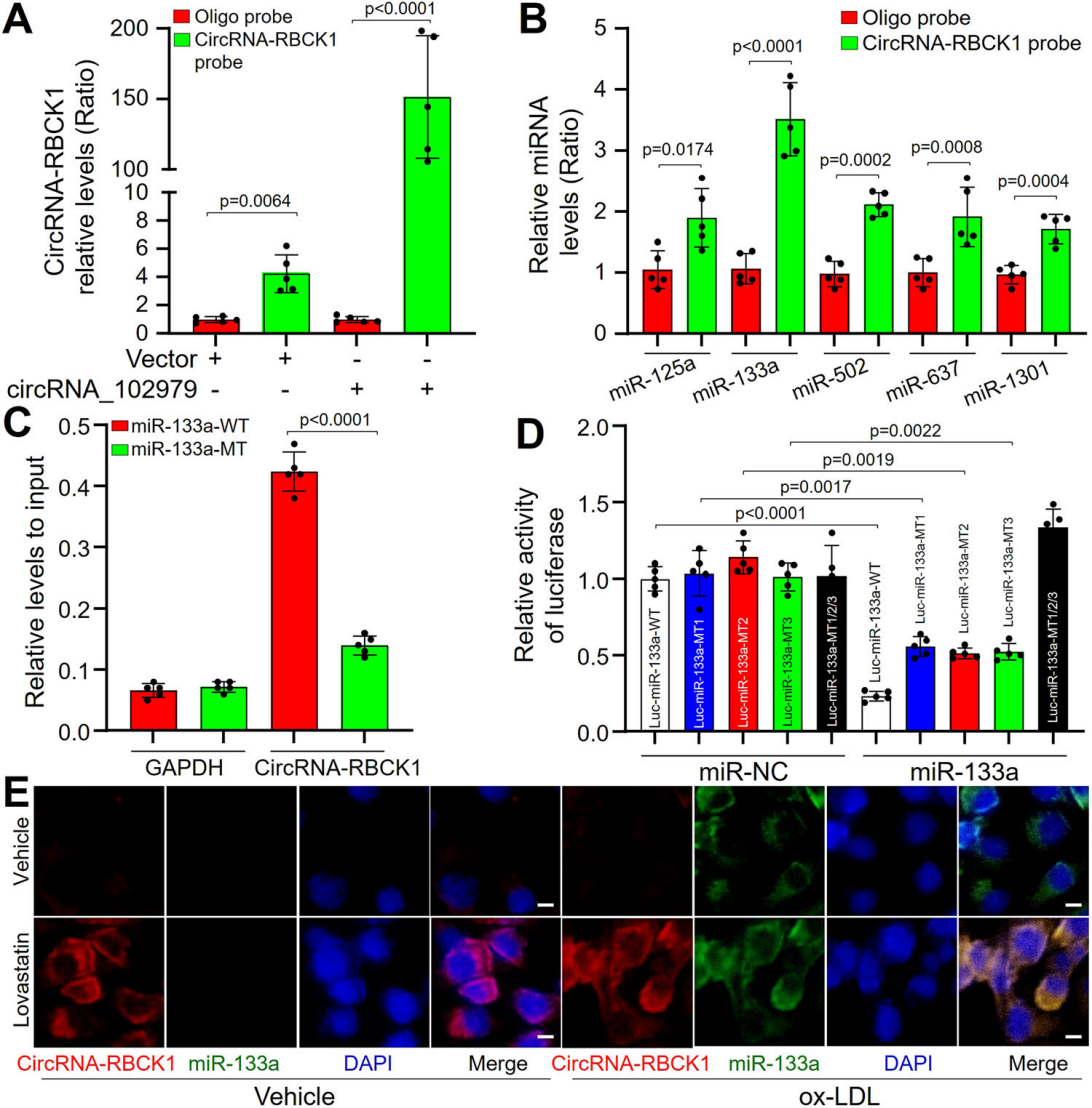
CircRNA-RBCK1 binds to miR-133a in human endocardium endothelial cells (EEC). **A** and **B** Human EECs were transfected with a plasmid expressing circRNA-RBCK1 for 24 h under ox-LDL (100 μg/ml). Total cell lysates were subjected to perform biotinylated-circRNA-RBCK1 pull-down assay followed by quantitative PCR analyses of circRNA-RBCK1 in (**A**) and top five candidate miRNAs predicted by CircNet database in (**B**). **C** The biotinylated wildtype (*WT*) or mutant (*MT*) miR-133a was, respectively, transfected into HEK293 cells with circRNA-RBCK1 overexpression. The levels of circRNA-RBCK1 were tested by quantitative PCR after streptavidin capture. **D** Plasmid of luciferase reporter construction containing circRNA-RBCK1 sequences with *WT* or mutated miR-133a binding sites (MT1, MT2, MT3, MT1/2/3) was co-transfected with miRNA negative control (miR-NC) or miR-133a in HEK293 cells. The luciferase activities in total cell lysates were assayed. **E** Human EECs were pretreated lovastatin (10 μM) for 2 h followed by ox-LDL (100 μg/ml) for 24h incubation. FISH was conducted to determine the co-location between circRNA-RBCK1 and miR-133a in human EECs. Scale bar, 5 µM. Red, circRNA-RBCK1; Green, miR-133a; Blue, nucleus. *N* = 5 per group in (**A–D**). Representative microscopy image was obtained from five independent experiments in (**E**). A one-way ANOVA followed by Tukey *post-hoc* tests was used to determine *P* value in **A** and **D**. A two-sided unpaired Student’s *t* test was used to determine *P* value in (**B**) and (**C**). Data are presented as mean ± SD. Source data are provided as a Source Data file.

To explore whether circRNA-RBCK1 binds these miRNAs in cardiac endothelial cells, 3′ terminal-biotinylated-circRNA-RBCK1 probe was designed to determine which miRNA potentially interacts with circRNA-RBCK1. The probe was verified to pull-down circRNA-RBCK1 in cells and circRNA-RBCK1 overexpression increased the pull-down efficiency (Fig. 2A). The quantitative PCR analysis revealed that miR-133a was the most abundantly pulled down by circRNA-RBCK1 probe in human EECs (Fig. 2B). To further consolidate the direct binding of miR-133a and circRNA-RBCK1, we utilized biotin-labeled miR-133a and its mutant mimics to pull-down circRNA-RBCK1 in EECs with circRNA-RBCK1 overexpression, the results showed wildtype *(WT*) miR-133a captured more circRNA-RBCK1 than the mutation (Fig. 2C).

Next, we cloned circRNA-RBCK1 luciferase reporter plasmid and performed reporter analysis in HEK293 cells. Co-transfection of miR-133a with luciferase reporter construction containing *WT* circRNA-RBCK1 sequence caused a significant inhibition of luciferase activity (Fig. 2D), compared to negative control. MiR-133a failed to suppress the activity of circRNA-RBCK1 luciferase reporter with a single mutated miR-133a binding site (*MT1, MT2, MT3*). Combined mutations of three site (*MT1/2/3*) further abolished the inhibitory effects of miR-133a on the activity of circRNA-RBCK1 luciferase reporter.

Because miR-133a is not positive but ectopically expressed under pathophysiological stimuli in endothelial cells^14^, we treated EECs with ox-LDL to induce miR-133a expressions. CircRNA-RBCK1 and miR-133a were co-localized in ox-LDL-treated EECs if they were pretreated with lovastatin (Fig. 2E). Further, enforced expression of miR-133a deceased GTPCH1 mRNA, BH4 content and NO production, but increased ROS generations (Supplementary Fig. 1C–F), proving that miR-133a suppresses GTPCH1 to induce eNOS uncoupling in cardiac endothelial cells.

### CircRNA-RBCK1 deficiency abolishes lovastatin-induced GTPCH1 upregulation and eNOS recoupling in EECs

**Fig. 3:**
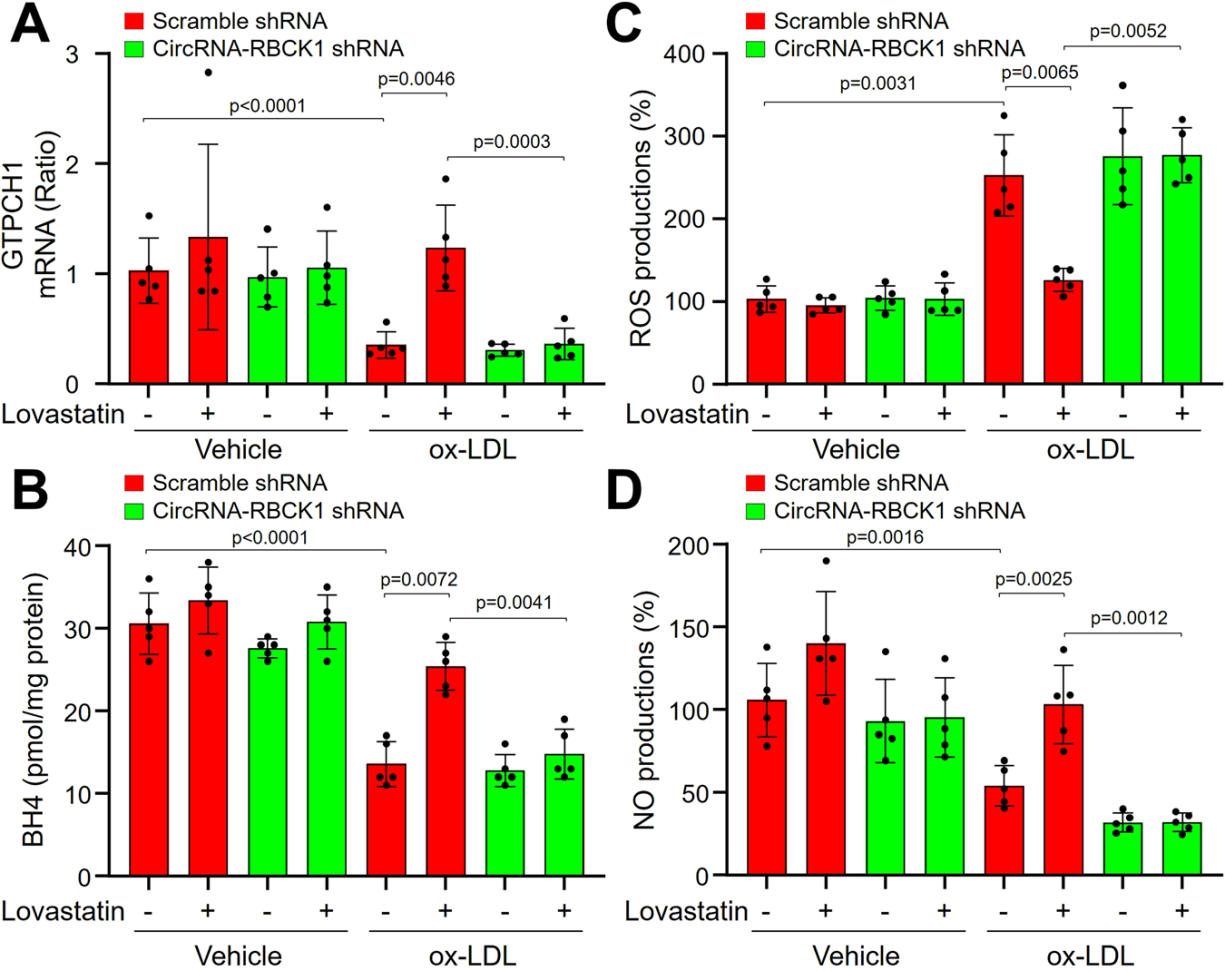
Lovastatin via circRNA-RBCK1 suppresses the function of miR-133a in human endocardium endothelial cells (EEC). Human EECs infected with lentivirus harboring scramble shRNA or circRNA-RBCK1 shRNA for 48 h were incubated with ox-LDL (100 μg/ml) plus lovastatin (10 μM) for 24 h. Cells were harvested to assay (**A**) GTPCH1 mRNA by quantitative PCR, (**B**) BH4 contents by HPLC, and (**C**) ROS productions by DHE/HPLC, and (**D**) NO levels by DAF/HPLC. *N* = 5 per group. A one-way ANOVA followed by Tukey *post-hoc* tests was used to determine *P* value in (**A**–**D**). Data are presented as mean ± SD. Source data are provided as a Source Data file.

We next infected EECs with lentivirus harboring specific shRNA to repress circRNA-RBCK1 expression (Supplementary Fig. 3A). As expected, lovastatin dramatically increased GTPCH1 mRNA and NO generations, but decreased BH4 levels and ROS productions in ox-LDL-treated EECs expressing scramble shRNA (Fig. 3A–D). As expected, these effects of lovastatin were abolished by circRNA-RBCK1 knockdown.

### AP-2α functions as a transcriptional factor of circRNA-RBCK1

**Fig. 4:**
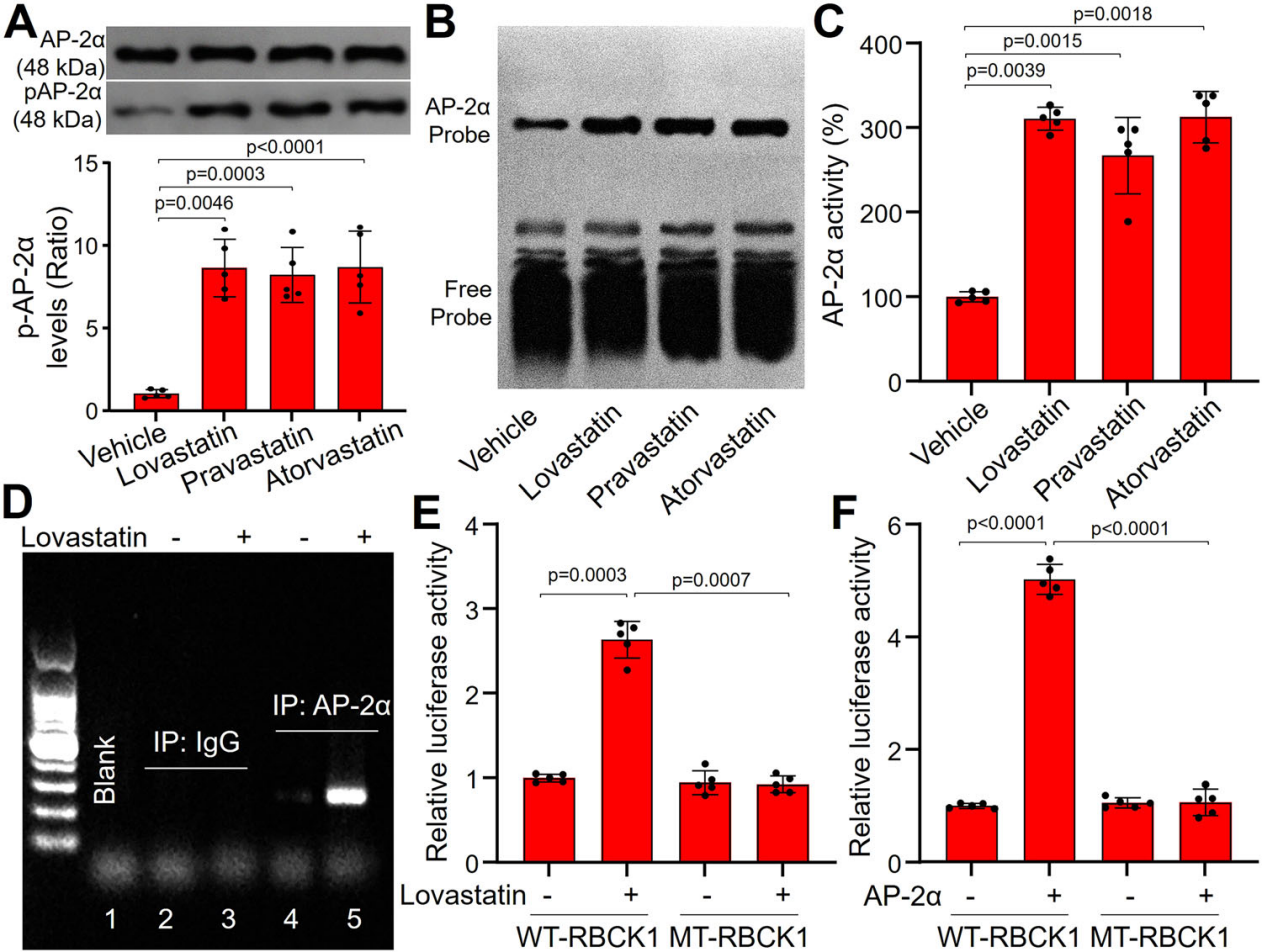
AP-2α is a transcriptional factor of circRNA-RBCK1 in human endocardium endothelial cells (EEC). **A**–**C** Human EECs were treated with lovastatin (10 μM), pravastatin (20 μM), and atorvastatin (10 μM) for 24 h. The protein levels of phosphorylated AP-2α at serine 219 (pAP-2α) and total AP-2α were measured in (**A**). The transcriptional activity of AP-2α was assayed by EMSA in (**B**) and (**C**). **D** Human EECs were treated with lovastatin (10 μM, 24 h). Cells were used for the detection of the affinity of AP-2α to RBCK1 gene promoter by ChIP assay. The representative image was obtained from five independent experiments. **E** and **F** HEK293 cells transfected with plasmid of luciferase reporter construction containing wildtype (WT) or mutant (MT) RBCK1 promoter were treated with lovastatin (10 μM, 24h) in (**E**) or co-transfected with AP-2α cDNA in (**F**). *N* = 5 per group. A one-way ANOVA followed by Dunnett test was used to determine *P* value in (**A**) and (**C**). A one-way ANOVA followed by Tukey *post-hoc* tests was used to determine *P* value in (**E**) and (**F**). Data are presented as mean ± SD. Source data are provided) as a Source Data file.

To identify how statins increase circRNA-RBCK1 gene transcription, we performed a proper bioinformatic analysis to investigate which transcriptional factor is likely to regulate circRNA-RBCK1 expression (http://jaspar.genereg.net). The score of AP-2α is the highest among 232 candidates (Supplementary Data 2), which is selected for this study. Three statins, such as lovastatin, pravastatin, and atorvastatin, increased AP-2α serine 219 phosphorylation in human EECs (Fig. 4A). EMSA analysis indicated that AP-2α transcriptional activities were also increased by statins (Figs. 4B, C).

We next performed a nucleotide sequence analysis of RBCK1 gene promoter to determine how AP-2α regulates circRNA-RBCK1 gene expression. The results revealed a potential AP-2α binding site (GCCGGAGGC) in the 5’ flanking region of human RBCK1 gene promoter (Supplementary Fig. 3B). ChIP assay indicated that lovastatin increased the binding of AP-2α to RBCK1 gene promoter (Fig. 4D).

To verify the specific interaction between AP-2α protein and RBCK1 gene promoter, we cloned luciferase reporter plasmid containing *WT* RBCK1 gene promoter (WT-RBCK1) or mutant of RBCK1 gene promoter which GCCCTGCGGC is replaced by ATTCTGCAAT (MT-RBCK1). As indicated in Fig. 4E, F, lovastatin or AP-2α overexpression increased the luciferase activity of the WT-RBCK1 promoter in HEK293, but not in the MT-RBCK1 promoter.

### AP-2α is indispensable for lovastatin-increased circRNA-RBCK1 gene expression in cardiac endothelial cells

**Fig. 5:**
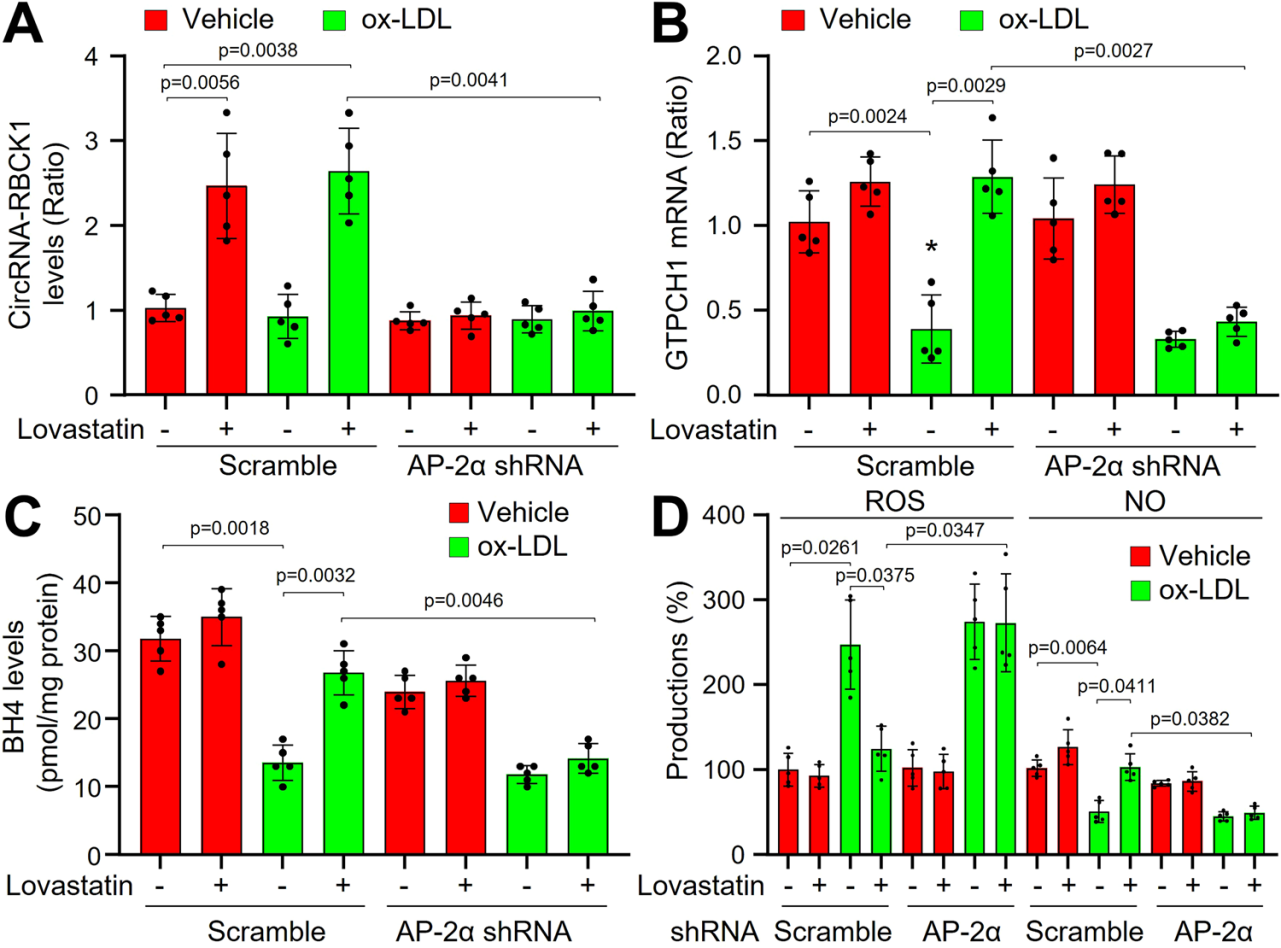
Lovastatin upregulates circRNA-RBCK1 to recouple eNOS in human endocardium endothelial cells (EEC), which is AP-2α dependent. Human EECs were infected with lentivirus expressing scramble shRNA or AP-2α shRNA for 48 h followed by incubation with lovastatin (10 μM) for 24 h in presence or absence of ox-LDL (100 μg/ml). Cells were harvested to assay the levels of circRNA-RBCK1 in (**A**) and GTPCH1 mRNA in (**B**) by quantitative PCR, BH4 contents by HPLC in (**C**), ROS productions by DHE/HPLC, and NO levels by DAF/HPLC in (**D**). *N* = 5 per group. A one-way ANOVA followed by Tukey *post-hoc* tests was used to determine the *P* value in this figure. Data are presented as mean ± SD. Source data are provided as a Source Data file.

It is reasonable to investigate if statins activate the GTPCH1/BH4/eNOS signaling via AP-2α. To this point, we infected EECs with lentivirus expressing AP-2α shRNA to block AP-2α gene expression (Supplementary Fig. 3C). Lovastatin increased circRNA-RBCK1 gene expression in ox-LDL-treated EECs expressing scramble shRNA (Fig. 5A–D). While, lovastatin did not increase circRNA-RBCK1 expression in EECs infected with lentivirus harboring AP-2α shRNA. Lovastatin significantly increased GTPCH1 mRNA levels and BH4 contents, and recoupled eNOS in ox-LDL-treated EECs expressing scramble shRNA but not AP-2α shRNA. Further, all phenomena produced by statins in human EECs were replicative in human MCECs (Supplementary Fig. 4A–E).

### Lovastatin ameliorates HFpEF phenotypes and increases endocardial circRNA-RBCK1 expression in mice

**Fig. 6:**
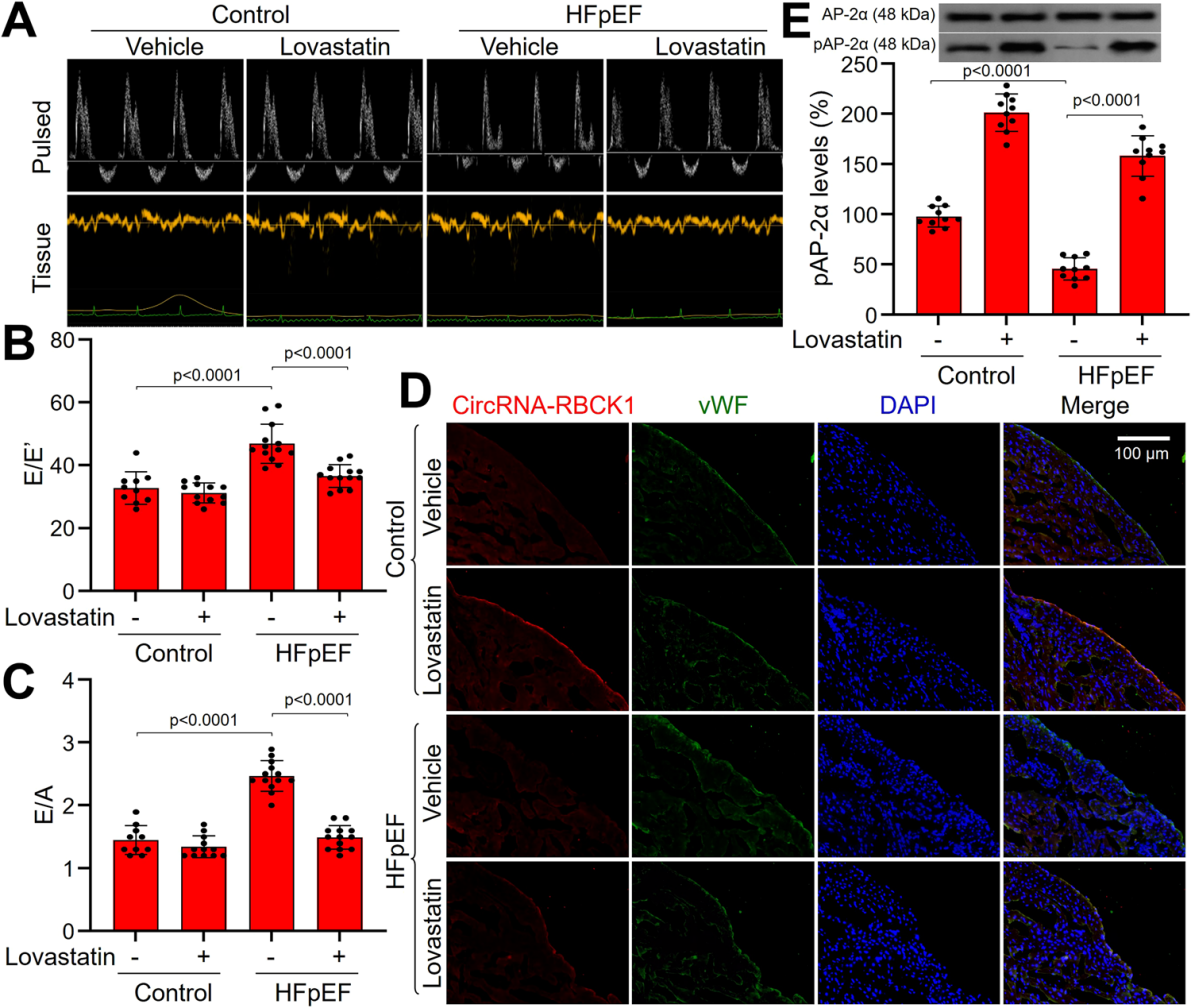
Lovastatin improves diastolic function in HFpEF mice. The protocols and experimental designs were described in Supplementary Fig. 5A. **A**–**C** Representative pulsed-wave Doppler (top) and tissue Doppler (bottom) tracings were shown in (**A**). The values of E/E′ in (**B**) and E/A in (**C)** were presented. **D** Hearts isolated from mice were subjected to perform FISH analysis of circRNA-RBCK1 gene expression in the endocardium. Red, circRNA-RBCK1; Green, vWF; Blue, nucleus. Representative microscopy image was obtained from ten mice in each group. **E** Left ventricle wall was isolated to determine pAP-2α level by western blot. In (**B**) and (**C**), *N* = 10 (control), *N* = 12 (control + lovastatin), *N* = 13 (HFpEF and HFpEF + lovastatin), a one-way ANOVA followed by Scheffe tests were used to determine the *P* value. In (**E**), *N* = 10 per group, a one-way ANOVA followed by Tukey *post-hoc* tests was used to determine the *P* value. Data are presented as mean ± SD. Source data are provided as a Source Data file.

Due to the protective effects of statins on cardiac endothelial cells, we next tested whether statins perform a therapeutic action in diastolic dysfunction in HFpEF. As indicated in Supplementary Fig. 5A, lovastatin was given to *WT* mice, which were followed by HFD plus AngII for 12 consecutive weeks to mimic clinical HFpEF^24^. HFD induced body weight gains, whereas AngII infusion raised both systolic and diastolic blood pressures (Supplementary Table 1). Longitudinal echocardiographic evaluation revealed persistent preservation of the left ventricular ejection fraction (LVEF) in all groups (Supplementary Fig. 5B and Supplementary Table 2). Mice exposed to HFpEF inducers manifested increased ratio of E wave to E′ wave on mitral tissue Doppler (E/E′) as well as increased early (E) wave to atrial (A) wave ratio (E/A) on mitral pulse Doppler, both indicative of diastolic dysfunction (Fig. 6A–C). Cardiac hypertrophy (HE staining and HW/TL) and fibrosis (Masson staining) were observed in mice treated with HFD plus AngII (Supplementary Figs. 5B, 6A). Consistent with the elevated filling pressures, mice exposed to HFD plus AngII uniquely exhibited a solid increase in lung weight (LW) and a reduced exercise capacity (Supplementary Fig. 6B, C), indicative of a preclinical surrogate for heart failure. In mice exposed to HFD plus AngII, lovastatin administration dramatically ameliorated these HFpEF phenotypes, compared to vehicle-treated HFpEF mice.

We next examined the effects of lovastatin on the AP-2α/circRNA-RBCK1 signaling in vivo. Similar to the in vitro results, lovastatin increased circRNA-RBCK1 expression in the endocardium (Fig. 6D), AP-2α phosphorylation in the heart (Fig. 6E), GTPCH1 mRNA level and BH4 content in the heart (Supplementary Fig. 6D, E).

We also compared the up-regulative effects of lovastatin on circRNA-RBCK1 gene expression between cardiac endothelial cells and vascular endothelial cells in vivo. As presented in Supplementary Fig. 7A–C, lovastatin upregulated circRNA-RBCK1 gene expressions in both cardiac endothelial cells and vascular endothelial cells in vivo. However, the up-regulative effects in cardiac endothelial cells were much stronger than vascular endothelial cells, consistent with the in vitro observations (Fig. 1C).

### Tamoxifen-induced endothelial cell-specific AP-2α gene knockout eliminates the beneficial effects of lovastatin on HFpEF mice

**Fig. 7:**
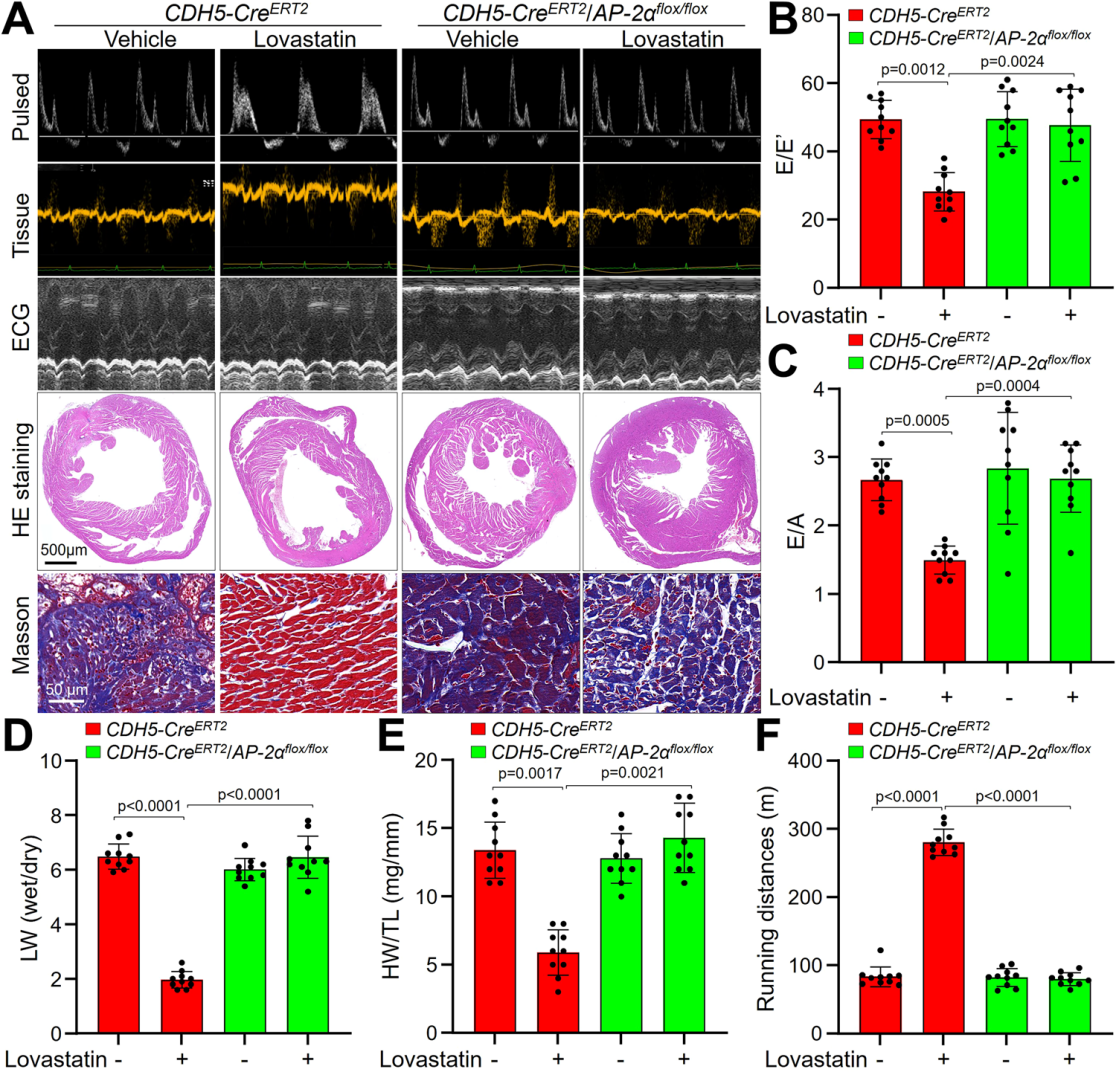
Tamoxifen-induced endothelial cell-specific AP-2α knockout eliminates the effects of lovastatin in HFpEF mice. The protocols were described in Supplementary Fig. 8C. **A**–**C** Representative pictures of pulsed-wave Doppler, tissue Doppler, M-mode echocardiographic tracings, HE staining, and Masson staining. Representative microscopy images of HE staining and Masson staining in (**A**) were obtained from ten mice in each group. The values of E/E′ in (**B**) and E/A in (**C**) were presented. **D**–**F** Ratio between wet and dry lung weight (LW) in (**D**), the ratio of heart weight to tibia length (HW/TL) in (**E**), running distance during exercise exhaustion test in (**F**) were calculated. *N* = 10 in per group. A one-way ANOVA followed by Tukey *post-hoc* tests was used to determine the *P* value in (**B**–**F**). Data are presented as mean ± SD. Source data are provided as a Source Data file.

We next generated *AP-2α*^*flox/flox*^/CDH5-Cre-ERT2 mouse to determine the in vivo roles of endothelial AP-2α activation in statins-improved diastolic functions (Supplementary Fig. 8A). *AP-2α*^*flox/flox*^/CDH5-Cre-ERT2 mouse was injected with tamoxifen to induce endothelium-specific AP-2α gene knockout (Supplementary Fig. 8B), which was followed by lovastatin administration prior to hyperlipidemia and hypertension (Supplementary Figs. 8B, C and Supplementary Table 3). Under HFpEF, the protective effects of lovastatin on diastolic functions (Fig. 7A–C and Supplementary Table 4), cardiac hypertrophy and fibrosis (Figs. 7A, D, E), and exercise capacity (Fig. 7F) were observed in tamoxifen-injected CDH5-Cre-ERT2 mice, but not in tamoxifen-injected *AP-2α*^*flox/flox*^/CDH5-Cre-ERT2 mice. Further, lovastatin increased circRNA-RBCK1 gene expressions in endocardium, GTPCH1 mRNA, and BH4 content in hearts isolated from tamoxifen-injected CDH5-Cre-ERT2 mice, rather than tamoxifen-injected *AP-2α*^*flox/flox*^/CDH5-Cre-ERT2 (Supplementary Fig. 9A–C).

### AAV9-mediated circRNA-RBCK1 gene knockdown abolishes lovastatin-improved diastolic functions in HFpEF mice

**Fig. 8:**
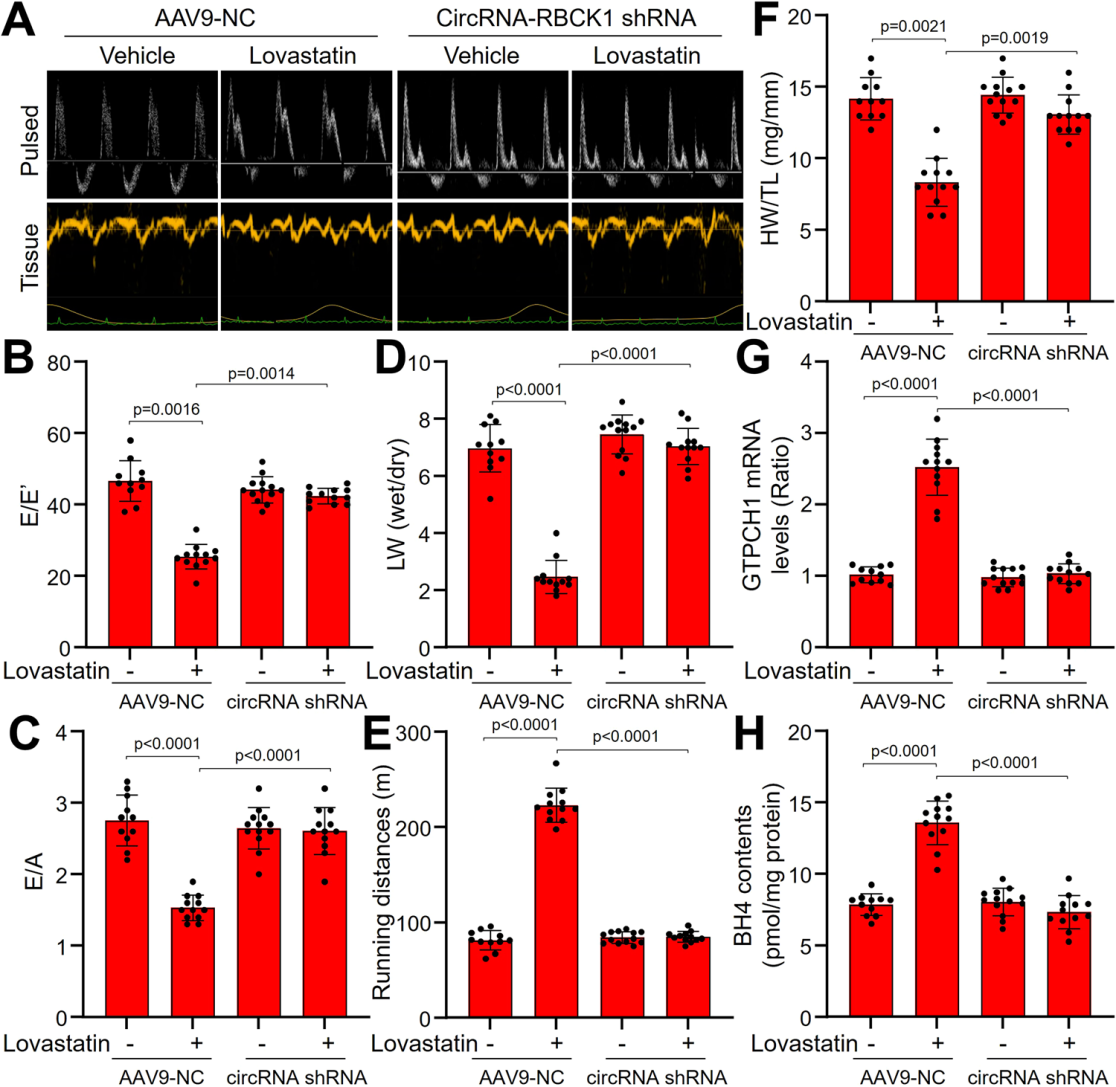
AAV9-mediated circRNA-RBCK1 gene knockdown abolishes the effects of lovastatin in HFpEF mice. The protocols and experimental designs were described in Supplementary Fig. 10A. **A**–**C** Representative pulsed-wave Doppler (top) and tissue Doppler (bottom) tracings were shown in (**A**). The values of E/E′ in (**B**) and E/A in (**C**) were presented. **D**–**F** Ratio between wet and dry lung weight (LW) in (**D**), running distance during exercise exhaustion test in (**E**), and the ratio of heart weight to tibia length (HW/TL) in (**F**) were calculated. **G** and **H** Left ventricle wall was isolated to determine GTPCH1 mRNA by quantitative PCR and BH4 contents by HPLC. *N* = 11 (AAV9-NC alone), *N* = 12 (AAV9-NC + lovastatin), *N* = 13 (circRNA shRNA alone), *N* = 12 (circRNA shRNA + lovastatin. A one-way ANOVA followed by Scheffe tests was used to determine the *P* value in (**B**–**H**). Data are presented as mean ± SD. Source data are provided as a Source Data file.

We next questioned whether gene knockdown of circRNA-RBCK1 can modulate the effects of statins on diastolic functions in vivo. To this end, loss-function of circRNA-RBCK1 was induced by infecting mice with AAV9 harboring circRNA-RBCK1 shRNA followed by lovastatin treatment and HFpEF induction (Supplementary Fig. 10A). AAV9 expressing circRNA-RBCK1 shRNA extremely inhibited circRNA-RBCK1 gene expression in endocardium (Supplementary Fig. 10B), but had no effects on the plasma levels of glucose and lipids in all mice (Supplementary Table 5). Lovastatin prevented diastolic dysfunctions (Fig. 8A–C and Supplementary Table 6), exercise capacity and LW (Figs. 8D, E), cardiac hypertrophy and fibrosis (Fig. 8F and Supplementary Fig. 11) in HFpEF mice if infected with AAV9 expressing negative control shRNA, but not in HFpEF mice expressing circRNA-RBCK1 shRNA. Accordingly, lovastatin increased GTPCH1 mRNA (Fig. 8G) and BH4 content (Fig. 8H) in the hearts of HFpEF mice expressing scramble shRNA, rather than HFpEF mice with exogenous expression of circRNA-RBCK1 shRNA.

EECs and MCECs share common features as modulators of cardiac performance^8,23^. Thus, we finally tested the effects of lovastatin on MCECs in vivo. As shown in Supplementary Fig. 12, lovastatin dramatically upregulated the gene expressions of circRNA-RBCK1 and GTPCH1 in MCECs isolated from HFpEF mice, and did not affect miR-133a levels. Similarly, the up-regulative effects of lovastatin on circRNA-RBCK1 and GTPCH1 were abolished in MCECs isolated from mice with deficient of AP-2α or circRNA-RBCK1, supporting the concept that statins suppress miR-133a through the activation of AP-2α/circRNA-RBCK1 signaling in cardiac endothelial cells.

## Discussion

The major discovery of this study is that miR-133a is bound by circRNA-RBCK1 in endothelial cells. Endothelial ectopic expression of miR-133a, liking miR-199 as a member of the myo-miRNA family, is a common mechanism of endothelial dysfunction induced by multiple risk factors^14,25^. In this study, we further identified miR-133a as a target of circRNA-RBCK1, and ascertained that statins upregulated circRNA-RBCK1 to prevent cardiac endothelial dysfunction. Recent observational studies indicate that statins may reduce mortality in HFpEF patients, particularly in the absence of coronary arterial disease, but independently of lipid lowering^13,26,27^. Our results may explain the molecular mechanism whereby statins inhibit miR-133a through circRNA-RBCK1 in cardiac endothelial cells, and improves the outcome of HFpEF.

Another important finding is that AP-2α functions as a transcriptional factor of RBCK1 gene to induce circRNA-RBCK1 expressions in cardiac endothelial cells. Although RBCK1 gene is regulated by several transcriptional factors, such as HOXA1 etc.^28^, in this study, we reported that AP-2α directly regulates RBCK1/circRNA-RBCK1 gene transcriptions. AP-2α is involved in multiple biological dysfunctions, such as abdominal aortic aneurysm^29^. Thus, the identification of AP-2α as an RBCK1 transcriptional factor explores the novel roles of RBCK1 and circRNA-RBCK1 in the aspects related to AP-2α.

Endothelial cells are the most abundant cell type in the heart in terms of absolute numbers^30^ and are increasingly recognized as regulators of tissue homeostasis and function^31,32^. EECs and MCECs, in contrast to coronary artery endothelial cells, are in close proximity to adjacent cardiomyocytes, allowing for direct communications^23^. Upon this structure of the artery, vascular endothelium releases NO to induce vasorelaxation, called as endothelium-dependent relaxation. Previously, it was considered that microvascular endothelial dysfunction is a key step in the formation of HFpEF^9,33^. Here, we consider the heart as artery, and think NO deficiency from both MCECs and EECs contributes to diastolic dysfunction. This helps to understand the pathophysiology of HFpEF.

Prevention of HFpEF through treatment of risk factors is effective^34^, but once HFpEF is present, specific treatments are lacking. Except for empagliflozin^35^, drugs used in patient with heart failure, such as diuretics, ARB, and RAAS, have not been similarly beneficial in HFpEF and increased the survival rate of patients with HFpEF^8,36,37^. Our finding is clinically relevant not only because we identified a previously unrecognized pathophysiological mechanism of diastolic function, but also unveiled a new pharmacological effect of statins on cardiac function by increasing cardiac endothelium-derived NO generation.

There are some limitations. First, it is better to distinguish the contributions among EECs, MCECs, and CAECs because they affect cardiac function in different ways^38^. However, there is no advanced technique to establish cardiac endothelial cells specific knockout mice. Second, due to the pre-use of lovastatin in this study, the therapeutical effects of statins on HFpEF need further investigations. Third, the potential impact of sex difference on animal model was not identified. Joseph A. Hill et al. have reported that female sex is protective in a preclinical model of HFpEF^39^. As a result, we only used male mice in this study. Fourth, considering statins could activate AP-2α, it is possible that other AP-2α regulated genes, but not circRNA-RBCK1, play critical roles in statins-prevented HFpEF. Finally, the effects of lovastatin on HFpEF clinical features show some contradictions, such as left ventricular remodeling and blood pressure. We thought that this discrepancy may attribute to the heterogeneous clinical manifestations of HFpEF^5,40^.

In summary, statins activate AP-2α to upregulate circRNA-RBCK1 gene expression to suppress miR-133a in cardiac endothelial cells. In this way, statins improves diastolic function to prevent HFpEF through GTPCH1/BH4/eNOS signaling. Therefore, the study will open new avenue to investigate the roles of AP-2α and circRNA-RBCK1 in cardiac endothelial dysfunction and also provide some insights of drug design for HFpEF patients by targeting AP-2α or circRNA-RBCK1, such as lovastatin.

## Methods

### Antibodies and reagents

Primary antibodies against AP-2α (#3208, dilution: 1:1000), vWF (#65707, dilution: 1:1000), and GAPDH (#5173, dilution: 1:1000) were obtained from Cell Signaling Technology (Boston, MA, USA). Primary GTPCH1 antibody (#ab307507, dilution: 1:1000) was from Abcam. Primary phosphorylated AP-2α at serine 219 antibody was generated by Genscript Company (dilution: 1:1000) as we described previously^29^. HRP-conjugated Affinipure Goat Anti-Rabbit IgG (H + L) (SA00001-1, dilution: 1:5000) were from Proteintech. Lovastatin (#438186), pravastatin (#1554206), atorvastatin (#524403), angintensin II (#05-23-0101), dihydroethidium (#309800), diaminofluorescein (#D224), and oxidized low-density lipoprotein (#AB3230) were purchased from Sigma-Aldrich Company (USA). High fat diet was purchased from Research Diet (D12492). Commercial kits for determinations of glucose, cholesterol, and triglyceride, LDL-C, and HDL-C were purchased from Jian-Cheng Bioengineering Institute (Nanjing, China). All drug concentrations are expressed as working concentrations in the buffer.

### Generation of *AP-2α*^*flox/flox*^ mice

Targeting vectors to generate *AP-2α*^*flox/flox*^ mice were constructed using a bacterial artificial chromosome (BAC, B6Ng01-349O17) recombineering (see Supplementary Fig. 8A for details). Bruce4 ES cells^41^ were electroporated with the targeting vector and positive clones identified by PCR and southern blotting were injected into blastocysts from C57BL/6-Tyrc-2J mice. The resulting male chimaeric mice were bred to female C57BL/6-Tyrc-2J mice to obtain germline transmission. The FRT-Neo-FRT cassette was removed by crossing with Flp deleter mice, then the mice were backcrossed at least three additional times onto a C57BL/6 background.

### BAC modification

A homologous recombination-proficient E. coli strain (DY380) was used for the BAC recombineering^42^. The 1^st^ step of the process is the homologous recombination of the BAC using the modification cassette I. It occurs by crossing over between the homology arms and the genome in the BAC. In this case, recombination results in the incorporation of the modification cassette sequences into the genome to yield the modified BAC1 (mBAC1). The 2^nd^ step of the process is the homologus recombination of the mBAC1 using the modification cassette II. It occurs by a second homologous recombination event that occurs within the mBAC1. Recombination yields the precisely modified BAC2 (mBAC2), with the modification cassette II inserted at the correct position in the BAC. This modified BAC carries the loxP-3’ region for introduction to the HAC vector. In brief, overnight cultures containing the BAC were grown from single colonies, diluted 10-fold in LB medium, and grown to an optical density at 600 nm of 0.4–0.6 at 32 °C. Fifty milliliter cultures were then induced for the expression of recombineering factors by shifting the cells to 42 °C for 15 min followed by chilling on ice for 10 min. Cells were then centrifuged for 5 min at 5500 X g at 4 °C and washed with 10 mL of ice-cold 1 mM HEPES 2 times. Cells were then resuspended in 100 μL of ice-cold 1 mM HEPES and electroporated. Cell transformation was performed by electroporation of 1 μg linear DNA into 100 μL of ice-cold competent cells in cuvettes (0.1 cm) using a Bio-Rad gene pulser set at 1.75 kV, 25 μF with a pulse controller set at 200 ohms. One milliliter of SOC medium was added after electroporation. Cells were incubated at 32 °C for 1 h with shaking and spread on appropriate selective agar media.

### Animals and induction of a ‘two-hit’ model of HFpEF

Male *WT* mice were obtained from Beijing Huafukang Company (Beijing, China). The *CDH5-Cre*^*ERT2*^ transgenic mouse exhibits tissue-specific expression of an inducible Cre-ERT2 fusion protein, enabling tamoxifen-induced Cre recombinase activity in vascular endothelial cells^43^, and were obtained from Taconic Biosciences, Inc (Model #13073). *AP-2α*^*flox/flox*^*/CDH5-Cre*^*ERT2*^ mouse was generated by crossing *AP-2α*^*flox/flox*^ mice with *CDH5-Cre*^*ERT2*^ mice. Male *CDH5-Cre*^*ERT2*^*/AP-2α*^*flox/flox*^ mouse was injected with tamoxifen (2 mg/mouse) for five consecutive days to induce endothelium-specific AP-2α gene knockout. Male *CDH5-CRE*^*ERT2*^ mouse injected with tamoxifen serves as a control because of endothelial CreERT2 toxicity^44,45^.

To induce HFpEF as the comorbidities of hypertension and obesity^24^, mice received HFD administration plus AngII infusion at a rate of 0.2 mg/kg per day for 12 weeks using a miniosmotic pump (Alzet), as reported previously with minor modifications^46–48^. This study was carried out in strict accordance with the recommendations in the Guide for the Care and Use of Laboratory Animals of the National Institutes of Health. The animal protocol was reviewed and approved by the Animal Care and Use Committee, Qilu Hospital of Shandong University.

### Conventional echocardiography (ECG) and doppler imaging

Transthoracic ECG was performed using a VisualSonics Vevo 2100 system equipped with an MS400 transducer (Visual Sonics). LVEF and other indices of systolic function were obtained from short-axis M-mode scans at the midventricular level, as indicated by the presence of papillary muscles, in conscious, gently restrained mice. Anesthesia was induced by 5% isoflurane and confirmed by a lack of response to firm pressure on one of the hindpaws. During echocardiogram acquisition under body-temperature-controlled conditions, isofluorane was reduced to 1.0–1.5% and adjusted to maintain a heart rate in the range of 400–500P beats per min.

To assess diastolic function, E, and E′were measured. After measurement of the parasternal short-axis view, the sonographic probe was tilted 45 degrees to visualize the parasternal 4-chamber view. Mitral E waves were recorded with pulse-wave mode at the mitral valve opening. Mitral annulus movement, also known as E′ wave, was assessed from the medial mitral valve annulus with tissue velocity image mode.

Parameters collected include: A, peak Doppler blood inflow velocity across mitral valve during late diastole; mitral DT, mitral early filling deceleration time; E, peak Doppler blood inflow velocity across mitral valve during early diastole; E′, peak tissue Doppler of myocardial relaxation velocity at mitral valve annulus during early diastole; HR, heart rate; IVRT, isovolumic relaxation time; IVS,d, end-diastolic interventricular septal wall thickness; LVEF, left ventricular ejection fraction; LVID,d, left ventricular internal diastolic diameter; LVID,s, left ventricular internal systolic diameter; LVPW,d, left ventricular end-diastolic posterior wall; LVFS, left ventricular fractional shortening.

### Blood pressure measurement

Blood pressure was determined by a left carotid catheter method at the end of animal experiments^49^. Mice were anesthetized with a ketamine and xylazine mixture and placed under warm light (37 °C). A catheter was inserted into the left common carotid artery, with the aid of a dissecting microscope, to measure arterial blood pressure. For catheter insertion, the left common carotid artery was carefully exposed via a 0.5–1.0 cm midline incision in the ventral neck region. The tip of the artery toward the head was ligated with a suture, and the tip toward the heart was occluded with a microclip. A small cut was then made in the vessel wall using microscissors. A 60 -cm catheter (PE10) containing a sterile 10% heparin-90% saline solution was inserted into the artery a distance of 0.65 cm toward the thorax. The arterial clip was removed, and the catheter was tied in place. Blood was directed to a pressure transducer through the catheter to obtain computerized blood pressure measurements (AD instruments). The mice were allowed to recover and the systolic and diastolic blood pressures and heart rate were monitored for at least 30 min in conscious states.

### Exercise exhaustion test

After three days of acclimatization to treadmill exercise, an exhaustion test was performed in the experimental groups of mice. Mice ran uphill (20°) on the treadmill (Columbus Instruments) starting at a warm-up speed of 5 m/min for 4 minutes after which speed was increased to 14 m/min for 2 minutes. Every subsequent 2 min, the speed was increased by 2 m/min until the mouse was exhausted. Exhaustion was defined as the inability of the mouse to return to running within 10 s of direct contact with an electric-stimulus grid. Running time was measured and running distance was calculated.

### Generations of AAV9 and infection to mice

The adeno-associated virus 9 (AAV9) construction compassing cDNA (AAV9-TIE-cDNA) was generated by according to the manufacturers’ recommendations from Shanghai Genechem Co., Ltd. (Shanghai, China). The endothelial cell specific promoter is “pAAV-TIEp-EGFP-MCS-3Flag-SV40 PolyA”. Viruses were packaged and amplified in HEK293A cells and purified using CsCl_2_ banding followed by dialysis against 10 mM Tris-buffered saline with 10% glycerol. Titering was performed on HEK293 cells using the adeno-X Rapid Titer kit (BD Biosciences Clontech, PaloAlto, CA, USA) according to the manufacturer’s instructions.

### Animal experimental protocols

In the first part of the animal study (Supplementary Fig. 5A), male *WT* mice received lovastatin administration (4 mg/kg/day) one week prior to HFD plus AngII treatments for another 12 weeks. In the second part of the animal study (Supplementary Fig. 8C), a male *AP-2α*^*flox/flox*^/CDH5-Cre-ERT2 mouse was injected with tamoxifen (2 mg/mouse) or vehicle for five consecutive days to induce endothelium-specific AP-2α gene knockout. Then mice received lovastatin administration (4 mg/kg/day) one week prior to HFD plus AngII treatments for another 12 weeks. In the third part of the animal study (Supplementary Fig. 10A), male *WT* mice were infected with AAV9 expressing negative control (NC) shRNA or circRNA-RBCK1 shRNA via tail vein injection. One week later, mice were treated with lovastatin (4 mg/kg/day) one week prior to HFD plus AngII treatments for another 12 weeks. An injection of AAV9 was repeated once in 4 weeks. For in vivo infection, AAV9 were injected via tail vein in 100 µl of PBS containing 1 ×10^11^ IFUs of loaded virus per mouse. The concentration of DNA was 10 mg/kg. Before mice sacrificed, conventional ECG and Doppler imaging were assessed.

### Hematoxylin-eosin staining

Slides with section were placed in a metal staining rack and immersed in the filtered Harris Hematoxylin for 10 s. Then, the sections were incubated in EOSIN stain for 30 s. Dehydration was performed in ascending alcohol solutions (50%, 70%, 80%, 95% X 2, 100% X 2) followed by clearance with xylene (3-4 X) in Columbia staining jars. The slides were mount using Permount (xylene based).

### Masson’s trichrome staining

The heart was fixed with 4% paraformaldehyde overnight at room temperature, dehydrated sequentially through ethanol, butyl alcohol, and embedded in paraffin. Hearts were serially cut from the apex to the base perpendicular to the long axis. Transverse sections (4 μm) were stained with Masson’s trichrome. Images were acquired on Panoramic MIDI (3D HISTECH Inc., Hungary).

### Quantitative PCR

Total RNA was isolated using a TRIzol-based (Invitrogen) RNA isolation protocol. RNA was quantified by Nanodrop (Agilent Technologies), and RNA and miRNA quality were verified using an Agilent 2100 Bioanalyzer (Agilent Technologies). Samples required 260/280 ratios of more than 1.8, and sample RNA integrity numbers of more than 9 for inclusion. RNA was reverse transcribed using the TaqMan microRNA Reverse Transcription Kit (Applied Biosystems) according to the manufacturer’s instructions. Quantification of circRNA and mRNA was performed using an ABI PRISM7500 system, and miRNA concentrations were determined using an ABI PRISM7900 system (Applied Biosystems, Carlsbad, CA, USA). Before calculation using the ^ΔΔ^Ct method, the levels of GAPDH were used to normalize the relative expression levels of circRNA and mRNA, and the levels of small nuclear U6 were used to normalize the miRNA expression levels. Primers used for quantitative PCR were listed in Supplementary Table 7.

### Mutagenesis of miR-133a binding sites in circRNA-RBCK1

These reporter constructs were generated in two steps. First, a coding-region fragment containing the miR-133a binding sites was generated by PCR and cloned into the pMIR luciferase vector (Ambion) using SpeI and MluI cloning sites. Next, site-directed mutagenesis was performed, introducing three mutations into the binding site’s seed sequence in the 3′-UTR of circRNA-RBCK1. Subsequently, a DNA fragment containing the 3′-UTR with the mutant of miR-133 binding site (*MT1, MT2, or MT3*) was generated by PCR and cloned into pMIR vector, this time using MluI and HindIII sites. Again, site-directed mutagenesis was used to change all three binding sites within the seed sequence (*MT1/2/3*). All constructs were sequenced to confirm their identity.

### Plasmid transfection into HEK293 and reporter assays

The sequences were synthesized and constructed into the pmirGLO luciferase plasmid vector, and promoter sequences were integrated and inserted into the GV272 vector by Genechem company (Shanghai, China). The plasmid constructs were co-transfected in HEK293 cells with the pCMV β-gal plasmid and 50 nM each of chemically synthesized miRNA oligonucleotides (Applied Biosystems) by using lipofectamine 2000 (Invitrogen). Cells were harvested 48 h after transfection, and luciferase and β-galactosidase activities were measured. The luciferase assay was conducted using a dual luciferase reporter assay (Vazyme, China) according to the manufacturer’s instructions.

### Cell cultures and virus infection

Primary HUVECs, human EECs, human CAECs, human MCECs, and human AECs were obtained from Clonetics Inc. (Walkersville, MD, USA). Cells were grown in endothelial basal medium supplemented with 2% fetal bovine serum and penicillin (100 u/ml), and streptomycin (100 µg/ml). Cultured cells were used between passages 3 and 8. All cells were incubated in a humidified atmosphere of 5%CO_2_ + 95% air at 37 °C. When 70–80% confluent, the cells were treated with different agents. For HEK293 cells, cells were cultured in M200 medium supplemented with 2% fetal bovine serum and penicillin (100 u/ml), and streptomycin (100 µg/ml). For lentivirus infection, cells were infected with lentivirus expressing AP-2α shRNA or circRNA-RBCK1 shRNA from Shanghai Genechem Co., Ltd. (Shanghai, China) overnight in antibiotics-free medium supplemented with 2% FBS. The target sequence of CircRNA-RBCK1 shRNA is TCTTGCAGCAGTGGGTGATTG. The target sequence of AP-2α shRNA is TCCCAGATCAAACTGTAATTA. These targets were designed by VectorBuilder Inc. The cells were then washed and incubated in fresh medium for an additional 12 h before experiments.

### Isolations of myocardial capillary endothelial cells, endocardium endothelial cells, coronary arterial endothelial cells, aortic endothelial cells from mice

For isolations of mouse aortic endothelial cells and mouse endocardium endothelial cells, heart and aorta were rapidly excised from mice (Supplementary Fig. 7A). Then, left ventricle and the aorta were injected with PBS containing 0.1% collagenase for 15 min at 37 °C. After centrifugation at 250 g for 10 min, the cell pellets were collected for RNA extraction immediately.

For isolation of mouse myocardial capillary endothelial cells, the distal left anterior descending coronary artery was cannulated, and the epicardial and endocardial surfaces were removed (Supplementary Fig. 7B). The remaining myocardial tissue was digested in PBS containing 0.1% collagenase at 37 °C for 30 min, with gentle rotation. Digested tissue was pelleted at 250 g and resuspended in DMEM containing 20% BSA (Sigma) (w/v), then myelin fraction was separated by centrifugation at 1000 g for 10 min. The cell pellet was resuspended and filtered through a 70 µM nylon mesh and collected following centrifugation at 250 g. The cell pellets were collected for RNA extraction immediately.

For isolation of mouse coronary arterial endothelial cells, the remained heart tissues by cutting the distal left anterior descending coronary artery were injected with PBS containing 0.1% collagenase for 15 min at 37 °C through the aortic root (Supplementary Fig. 7B). After centrifugation at 250 g for 10 min, the cell pellets were collected for RNA extraction immediately.

### RNA extraction and sequencing data analysis

Total RNA from 6 samples was extracted. We have generally utilized 100 ng of RNA for library construction for MeRIP-circRNA sequencing. Briefly, the mRNA with polyA in the total RNA was enriched by Oligo-dT magnetic beads. The intact mRNA was then fragmented using an ultrasound machine. The segmented RNA was divided into two parts. One part was added to an m6A-capturing antibody to enrich the mRNA fragments containing m6A methylation (MeRIP-seq), and the other part was used as an Input to directly construct a conventional transcriptome sequencing library (circRNA-seq). The conventional sequencing library was constructed according to the transcriptome library construction process. Illumina Hiseq X Ten was used for high-throughput sequencing of the library. The circBase database and Circ2Traits were used to annotate the identified circRNA. Then, DESeq2 software (v1.14.1) was used for data standardization and differentially expressed circRNA screening (log2FC ≥ 1.5, p-value ≤ 0.05).

### Biotinylated RNA pull-down assay

The biotinylated-circRNA-RBCK1 probe was incubated with C-1 magnetic beads (Life Technologies, Carlsbad, CA, USA) to generate probe-coated beads, then incubated with sonicated HEK293 cells at 4 °C overnight, followed by eluted and quantitative RT-PCR. For miR-133a pulled down circRNA-RBCK1, HEK293 cells with circRNA-RBCK1 overexpression were transfected with biotinylated miR-33a mimics or mutant using Lipofectamine 2000. The cells were harvested, lysed, sonicated, and incubated with C-1 magnetic beads (Life Technologies, Carlsbad, CA, USA), followed by quantitative RT-PCR.

### Chromatin-immunoprecipitation assay for the binding of AP-2α and RBCK1 gene promoter

The binding between AP-2α and RBCK1 gene promoter was performed using a bioinformatic analysis (http://genexplain.com/transfac). According to the scores, ChIP assays were performed by using a ChIP-IT kit (Upstate, 17-295), according to the manufacturer’s protocol. 1 × 10^6^ cells were seeded on a 10 cm dish. Proteins were cross-linked to DNA by adding formaldehyde directly to the culture medium at a final concentration of 1% and incubating for 10 min at 37 °C. The cells were harvested in SDS lysis buffer and added protease inhibitors. Cell lysates were sonicated to shear DNA to lengths between 200 and 1000 bp. Sheared chromatin was precleared with protein G beads prior to incubation overnight at 4 °C with 4 µg of AP-2α antibody or IgG antibody. Purified, immunoprecipitated chromatin fragments from IP samples were subjected to PCR. PCR products were subjected to agarose gel electrophoresis and stained with ethidium bromide. The primer sequences were as follow: forward, 5’-attcatgtgcaaacggggc-3’, and reverse, 5’-aggcgacccggaggtagcatt-3’.

### Electrophoretic mobility shift assay for AP-2α activity

Subcellular fractions were prepared using NE-PER Nuclear and Cytoplasmic Extract kit (Cat78833) from PIERCE. EMSA were performed following the commercial kits. AP-2α kit (AY1002) is from Panomics Company.

### RNA fluorescence in situ hybridization

The RNA fluorescence in situ hybridization assay was performed by using a FISH kit (RiboBio, Guangzhou, China) according to the manufacturer’s guidelines. Cy3-labeled circRNA-RBCK1 probes and Dig-labeled locked nucleic acid miR-133a probes (Ribo-Bio, Guangzhou, China) were measured by the FISH kit, followed by visualized with a confocal microscopy.

### Immunofluorescence analysis

Sections were deparaffinized, rehydrated, and blocked with 5% normal serum. Incubate tissues with primary antibody for 1 h at room temperature or overnight at 4 ^o^C. After washing, incubate with fluorescence-conjugated secondary antibody for 45 minutes. Digital images were captured under a fluorescence microscopy. Quantitative analysis was performed by calculating fluorescence intensity using Alpha Ease FC software (version 4.0 Alpha Innotech).

### Western blotting

Tissues were homogenized on ice in cell-lysis buffer (20 mM Tris-HCl, pH 7.5, 150 mM NaCl, 1 mM Na_2_EDTA, 1 mM EGTA, 1% Triton, 2.5 mM sodium pyrophosphate, 1 mM beta-glycerophosphate, 1 mM Na_3_VO_4_, 1 µg/ml leupeptin) and 1 mM PMSF. Cell was lysated with cell-lysis buffer. The protein content was assayed by BCA protein assay reagent (Pierce, USA). 20 µg proteins were loaded to SDS-PAGE and then transferred to membrane. Membrane was incubated with a 1:1000 dilution of primary antibody, followed by a 1:2000 dilution of horseradish peroxidase- conjugated secondary antibody. Protein bands were visualized by ECL (GE Healthcare). The intensity (area X density) of the individual bands on Western blots was measured by densitometry (model GS-700, Imaging Densitometer; Bio-Rad). The background was subtracted from the calculated area.

### Detection of ROS

Cells were incubated with DHE (10 µM) for 30 min, homogenized, and subjected to methanol extraction. HPLC was performed using a C-18 column (mobile phase: gradient of acetonitrile and 0.1% trifluoroacetic acid) to separate and quantify oxyethidium (product of DHE and O_2_^-^) and ethidium (a product of DHE auto-oxidation). ROS level was determined by conversion of DHE into oxyethidine. To measure ROS production in the artery in situ, fresh frozen sections of MCA were isolated from mice, and were stained with 10 μM DHE for 30 min, rinsed, and observed by fluorescent microscopy. Results were quantified using BIOQUANT Image software.

### Detection of intracellular NO

NO production in culture cells was detected using the fluorescent probe. Briefly, before the end of treatment, 10 µM DAF was added to the medium and incubated for 30 min at 37 °C, then washing with PBS twice. The DAF fluorescent intensity was recorded by fluorescent reader at the wave of excitation (485 nm) and emission (545 nm).

### Measurement of BH4

Homogenates of aorta or cell lysates were suspended in distilled water containing 5 mM dithioerythrol, centrifuged at 12,000 g at 4 °C for 10 min, and then subjected to oxidation in acid or base. To 100 μl aliquot of supernatant, 20 μl of 0.5 M HCl and 0.05 M iodine were added for acidic oxidation, and 20 μl of 0.5 M NaOH plus 0.05 M iodine were added for basic oxidation. After incubation for 1 h in the dark at room temperature, 20 μl HCl was added to the basic oxidation only. All mixtures received 20 μl of 0.1 M ascorbic acid for the reduction of excess iodine. Samples were then centrifuged for 10 min at 12000 g at 4 °C. Biopterin concentrations were determined by HPLC with a PR-C18 column. Elution was at a rate of 1.0 ml/min of 50 mM potassium phosphate buffer, pH 3.0. Fluorescence was detected with an excitation at 350 nm and emission at 440 nm. BH4 concentrations were calculated as the difference in results from oxidation in acid and base.

### Measurements of blood glucose, cholesterol, and triglyceride

Blood glucose, homocysteine, cholesterol, triglyceride, LDL-C, and HDL-C were assayed by using commercial kits as recommend by the protocol.

### Statistical analysis

All quantitative results were expressed as mean ± SD. The normal distribution of data was tested by the Kolmogorov-Smirnov test before statistical comparisons, and the normality/equal variance was tested to determine whether ANOVA was appropriate. A one-way ANOVA followed by Tukey’s HSD test, Scheffe test or Dunnett test were used to multiple comparisons between two groups. Statistical analysis was conducted using IBM SPSS statistics 20.0 (IBM Corp., Armonk, NY, USA). *P* < 0.05 was considered significant. GraphPad Prism version 8 (GraphPad Software, San Diego, CA, USA, www.graphpad.com) was used to make figures.

### Reporting summary

Further information on research design is available in the Nature Portfolio Reporting Summary linked to this article.

### Supplementary information

Supplementary Information

Description of Additional Supplementary Files

Supplementary Data 1

Supplementary Data 2

Reporting Summary

### Source data

Source Data

## Data Availability

Data supporting the findings of this study are available within the article and its Supplementary Information files. Source data are provided in this paper. The data reported in this paper have been deposited in the OMIX, China National Center for Bioinformation / Beijing Institute of Genomics, Chinese Academy of Sciences (https://ngdc.cncb.ac.cn/omix: accession no.OMIX005995). Source data are provided in this paper.
