# Supplementary material for: Statins improve cardiac endothelial function to prevent heart failure with preserved ejection fraction through upregulating circRNA-RBCK1
Source: Nat Commun. 2024 Apr 5;15:2953. doi: 10.1038/s41467-024-47327-z (PMC10997751; doi:10.1038/s41467-024-47327-z)
Supplement: Supplementary file 1 — Supplementary Information [file 41467_2024_47327_MOESM1_ESM.pdf]

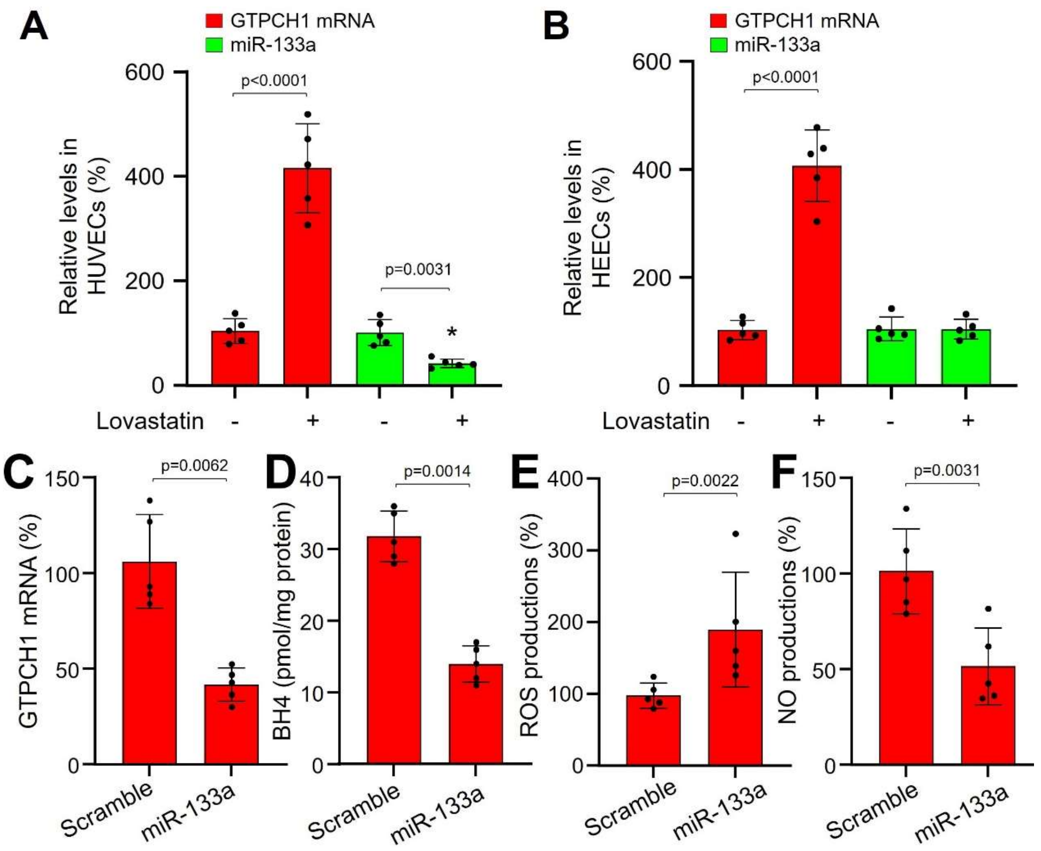

Supplementary Figure 1. Lovastatin increases GTPCH1 gene expression but has no effects on miR-133a expression in cardiac endothelial cells. (**A** and **B**) HUVECs in **A** and human EECs in **B** were pretreated lovastatin (10 μM) for 30 minutes followed by co-incubation with ox-LDL (100 μg/ml) for 24 hours. Total RNAs were extracted from cells to measure GTPCH1 mRNA and miR-133a expression using quantitative PCR. N = 5 per group. (**C-F**) Human EECs were infected with lentivirus harboring scramble microRNA or pre-miR-133a for 48 hours. Cells were subjected to detect GTPCH1 mRNA using quantitative PCR in **C**, BH4 contents by HPLC in **D**, ROS productions by DHE/HPLC in **E**, and NO levels by DAF/HPLC in **F**. An unpaired Student's *t* test was used to determine *P* value between two groups in this figure. Data are presented as mean ± SD. Source data are provided as a Source Data file.

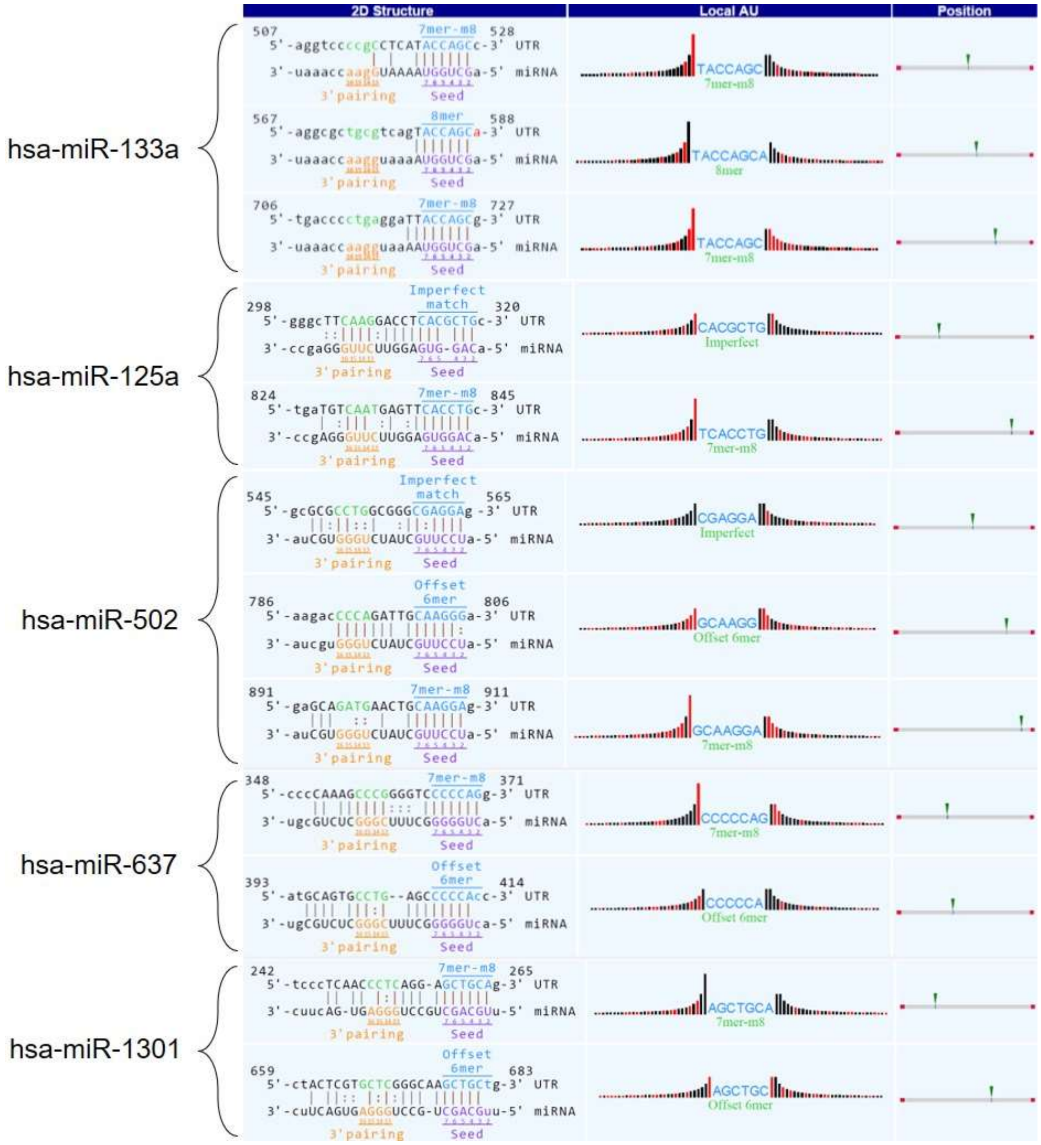

Supplementary Figure 2. Predictions of miRNAs targeted by circRNA-RBCK1 using CircNet database. Five miRNAs including miR-125a, miR-133a, miR-502, miR-637, and miR-1301 could potentially bind to the highly conserved target sites in the 3′-UTR of circRNA-RBCK1.

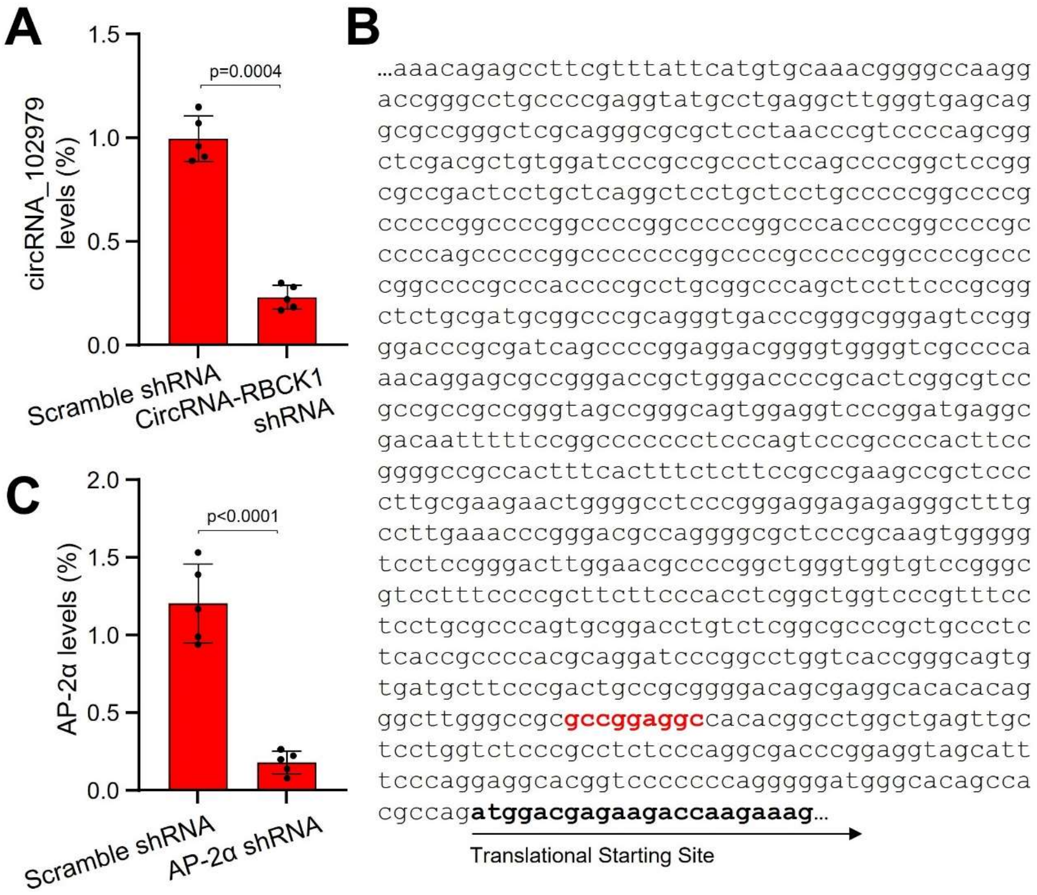

Supplementary Figure 3. Efficacy of lentivirus expressing shRNA in human endocardium endothelial cells (EECs). (**A**) Cultured human EECs were infected with lentivirus expressing scramble shRNA or circRNA-RBCK1 shRNA for 48 hours. Cells were harvested to assay the levels of circRNA-RBCK1 by quantitative PCR. The target sequence of CircRNA-RBCK1 shRNA is TCTTGCAGCAGTGGGTGATTG. (**B**) Interaction between AP-2α and human RBCK1 gene promoter. The binding site of AP-2α is marked in red (GCCGGAGGC). The bolds "**atggacgagaagaccaagaaag**" means the translational starting site of circRNA-RBCK1 gene. The promoter region as 1000 bp the upstream of ATG (translational site). (**C**) Human EECs were infected with lentivirus expressing scramble shRNA or AP-2α shRNA for 48 hours. Cells were harvested to assay the levels of AP-2α mRNA by quantitative PCR. The target sequence of AP-2α shRNA is TCCCAGATCAAACTGTAATTA. N = 5 per group. An unpaired Student's *t* test was used to determine *P* value in **A** and **C**. Data are presented as mean ± SD. Source data are provided as a Source Data file.

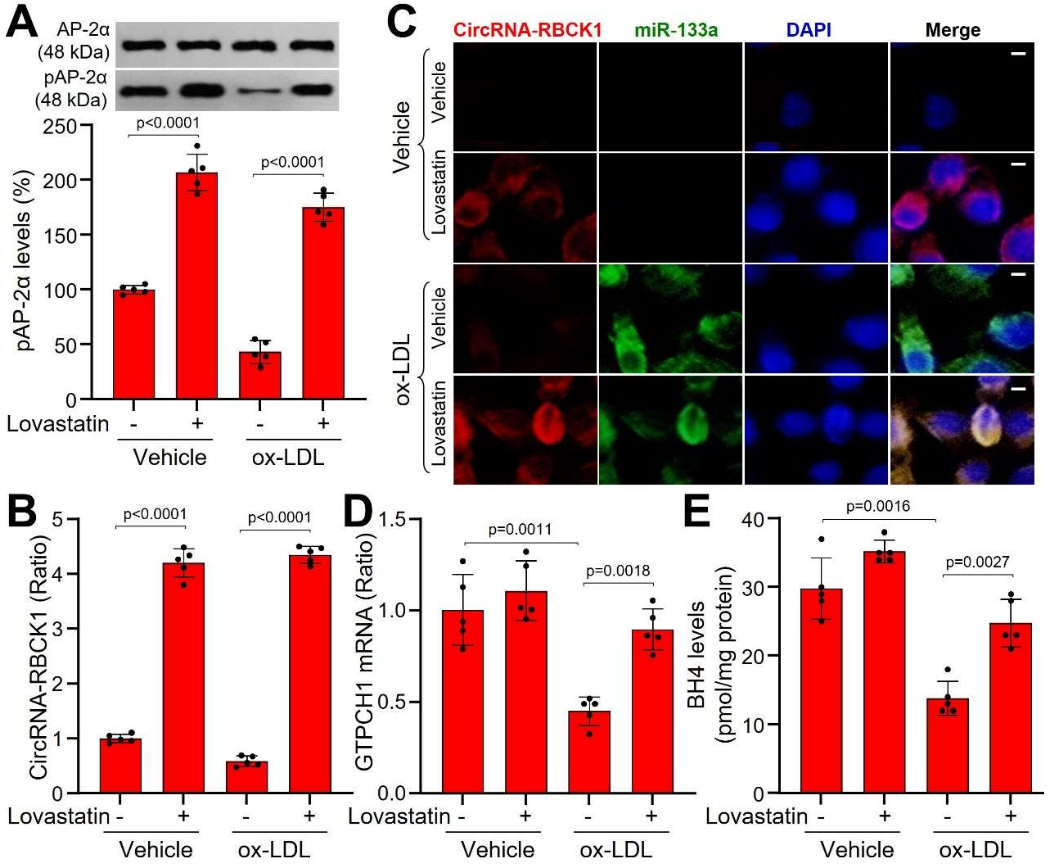

Supplementary Figure 4. CircRNA-RBCK1 binds to miR-133a in human myocardial capillary endothelial cells (MCEC). Human MCECs were pretreated with lovastatin (10 µM) for 2 hours followed by ox-LDL (100 µg/ml) for 24 hours. (**A** and **B**) Total cell lysates were subjected to determine pAP-2α level by Western blot in **A** and circRNA-RBCK1 gene expression in **B**. (**C**) FISH was conducted to determine the co-location between circRNA-RBCK1 and miR-133a in cells. Scale bar, 5 µM. Red, circRNA-RBCK1; Green, miR-133a; Blue, nucleus. Representative image was obtained from five independent experiments. (**D** and **E**) GTPCH1 mRNA by quantitative PCR in **D** and intracellular BH4 contents by HPLC in **E** were measured. N = 5 per group. A one-way ANOVA followed by Tukey *post-hoc* tests was used to determine *P* value in **A**, **B**, **D**, and **E**. Data are presented as mean ± SD. Source data are provided as a Source Data file.

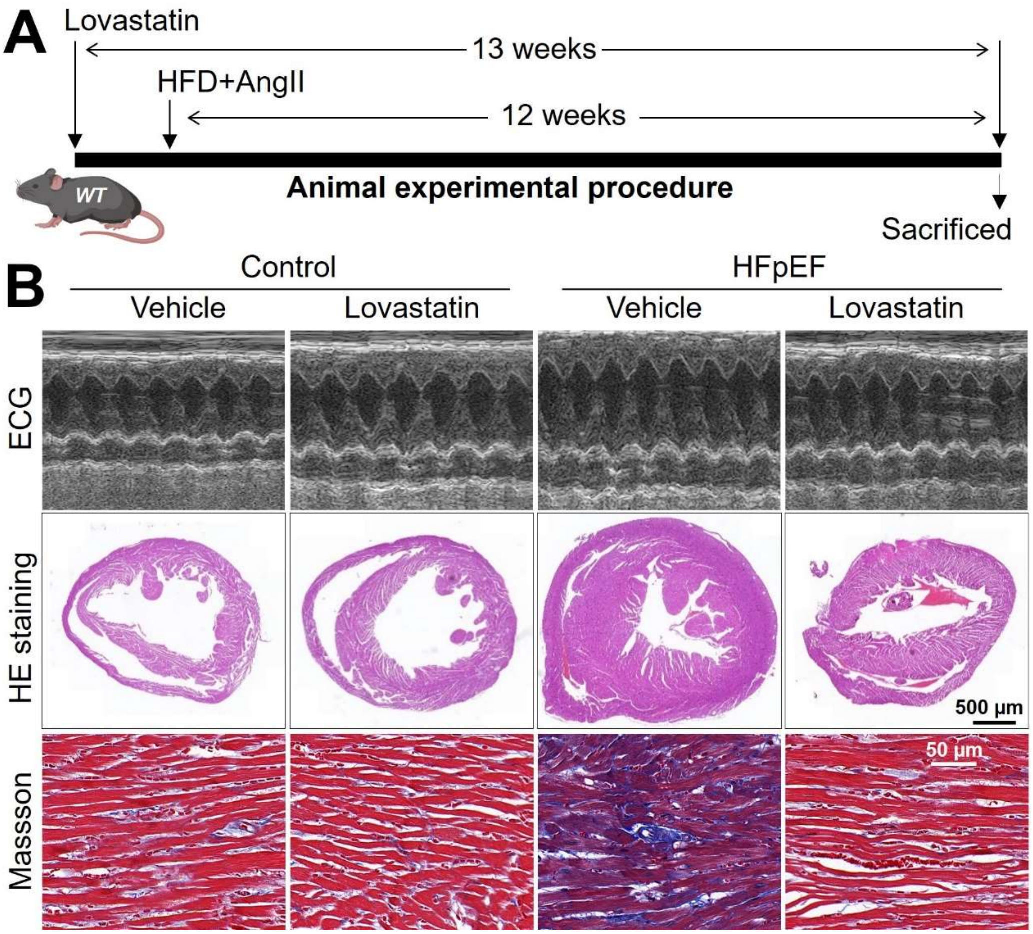

Supplementary Figure 5. Lovastatin inhibits cardiac hypertrophy and fibrosis in HFpEF mice induced by HFD plus AngII. (**A**) Graphical procedures of animal experiments. Mice received lovastatin administration (4 mg/kg/day). One week later, mice received HFD and AngII treatments for 12 consecutive weeks. Before sacrifice, conventional ECG and Doppler imaging were assessed. (**B**) Representative left ventricular M-mode echocardiographic tracings before mice were sacrificed. Hearts isolated from mice were subjected to perform HE staining and Masson staining. The representative pictures were shown. Parameters obtained from ECG were summarized in Supplementary Table 2. Adobe illustrator was used to create mouse image by Lin Chen.

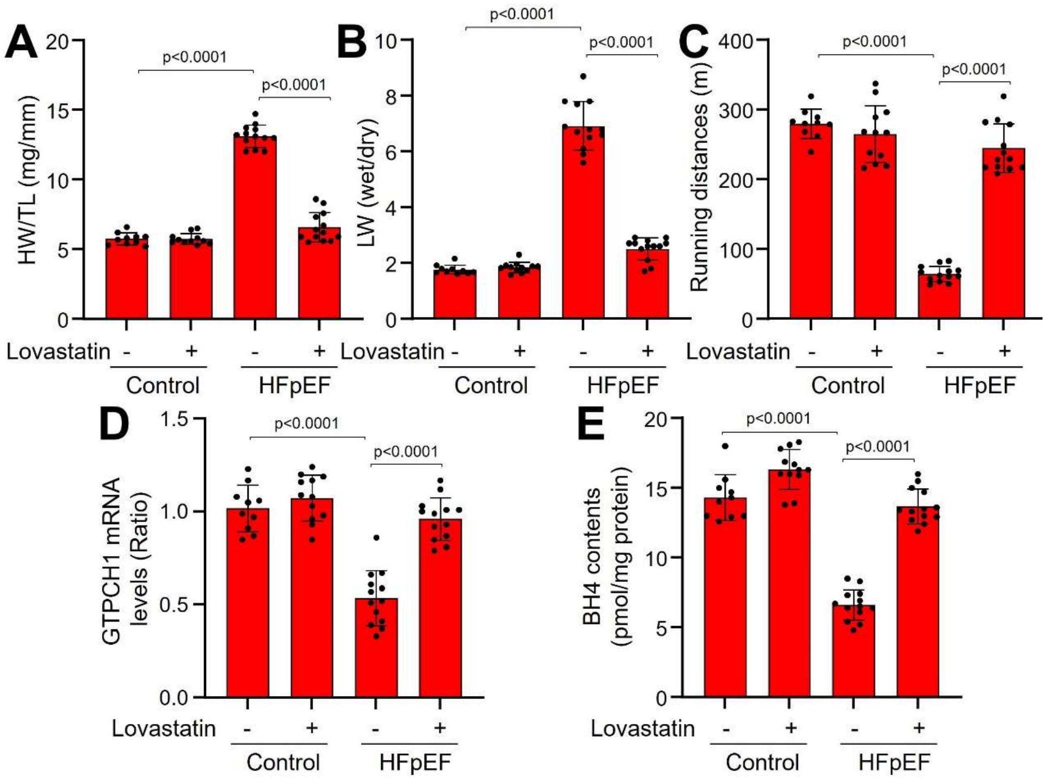

Supplementary Figure 6. Lovastatin improves HFpEF features and upregulates GTPCH1/BH4 in HFpEF mice. Graphical procedures of animal experiments were shown in Supplementary Figure 5A. (**A-C**) Ratio of heart weight to tibia length (HW/TL) in **A**, ratio between wet and dry lung weight (LW) in **B**, and running distance during exercise exhaustion test in **C** were calculated. (**D** and **E**) Left ventricle wall was isolated to determine GTPCH1 mRNA by quantitative PCR in **D** and BH4 contents by HPLC in **E**. N = 10 (control), N = 12 (control + lovastatin), N = 13 (HFpEF and HFpEF + lovastatin). A one-way ANOVA followed by Scheffe tests was used to determine *P* value in this figure. Data are presented as mean ± SD. Source data are provided as a Source Data file.

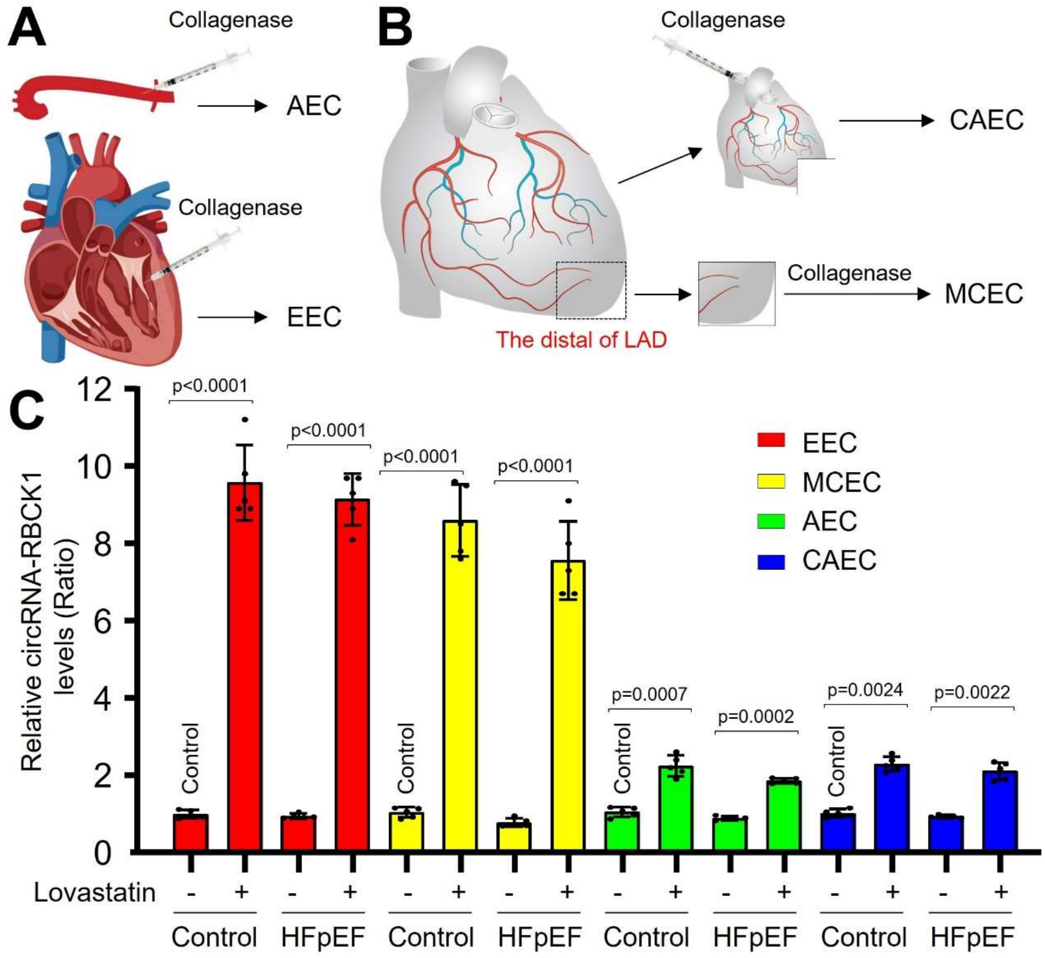

Supplementary Figure 7. The *in vivo* expressions of circRNA-RBCK1 in endothelial cells isolated from lovastatin-treated HFpEF mice. Graphical procedures of animal experiments were shown in Supplementary Figure 5A. (**A**) Isolations of endocardium endothelial cells (EEC) and aortic endothelial cells (AEC) from mice. (**B**) Isolations of myocardial capillary endothelial cells (MCEC) and coronary arterial endothelial cells (CAEC) according to coronary segmentation. (**C**) Total RNAs in primary endothelial cells were extracted immediately after isolations from mice and subjected to determine the levels of circRNA-RBCK1 using quantitative PCR. The circRNA-RBCK1 level in control group was setup as 1. N = 5 per group. A one-way ANOVA followed by Tukey *post-hoc* tests was used to determine *P* value in **C**. Data are presented as mean ± SD. Source data are provided as a Source Data file. Adobe illustrator was used to create heart image by Lin Chen.

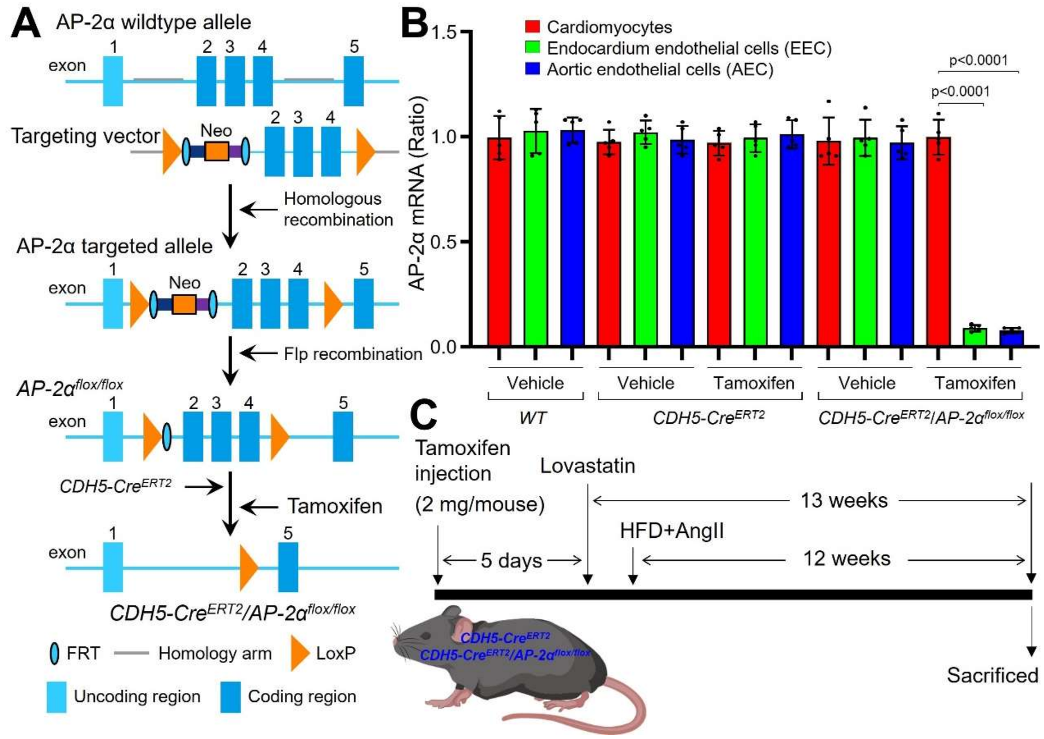

Supplementary Figure 8. The methodology of inducible endothelial cell-specific AP-2α gene knockout mouse generation and identification. (**A**) A bacterial artificial chromosome (BAC) clone containing the mouse AP-2α gene was modified to insert a loxP-FRT-Neo-FRT cassette 5' to exon 2 and a loxP site 3' to exon 4 to generate the targeting vector. These sites were selected to avoid disrupting conserved sequences, which are potential regulatory elements. Resolved BAC candidates were first screened by PCR. DNA from PCR positive clones was subsequently digested and separated by electrophoresis on a 1% agarose gel, and transferred onto a nylon membrane. The blot was hybridized with a radiolabeled DNA probe using southern blotting. *AP-2α$^{fl\text{-}Neo}$* mice were crossed with Flp delete mice to remove the Neo cassette to get *AP-2α$^{flox/flox}$*. *AP-2α$^{flox/flox}$* mice crossed with *CDH5-Cre^{ERT2}* mice to generate *CDH5-Cre^{ERT2}/AP-2α$^{flox/flox}$* mice. *AP-2α$^{flox/flox}$/CDH5-Cre^{ERT2}* mouse was injected with tamoxifen (2 mg/mouse) for five consecutive days to induce endothelium-specific AP-2α gene knockout. *CDH5-Cre^{ERT2}* mouse injected with tamoxifen serves as control mouse. (**B**) Primary cardiomyocytes, endocardium endothelial cells (EEC), and aortic endothelial cells (AEC) were isolated from *WT, CDH5-Cre^{ERT2}*, and *AP-2α$^{flox/flox}$/CDH5-Cre^{ERT2}* with or without tamoxifen injection. Total RNAs were

extracted immediately after isolation. The levels of AP-2α mRNA were measured using quantitative PCR. N = 5 per group. (**C**) The animal protocol of lovastatin therapy in endothelium-specific AP-2α gene knockout mice with HFpEF. After tamoxifen injection, *CDH5-Cre*^{ERT2} and *CDH5-Cre*^{ERT2}*/AP-2α*$^{flox/flox}$ mice received lovastatin administration (0.1%, approximately 4 mg/kg/day) for 13 consecutive weeks. HFpEF model was induced by feeding mice with HFD and AngII for 12 weeks. Before sacrifice, conventional ECG and Doppler imaging were assessed. Data are presented as mean ± SD. Source data are provided as a Source Data file. Adobe illustrator was used to create mouse image by Lin Chen.

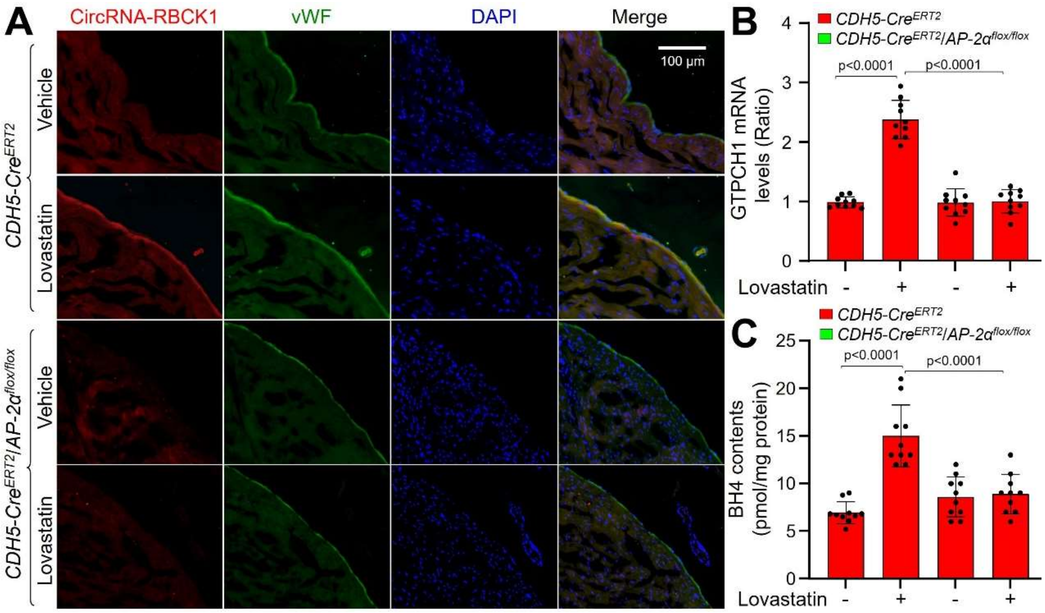

Supplementary Figure 9. Endothelial AP-2α is required for lovastatin to increase circRNA-RBCK1 gene expression in endocardium of HFpEF mice. Graphical procedures of animal experiments were shown in Supplementary Figure 8C. (**A**) Hearts isolated from mice were subjected to perform FISH analysis of circRNA-RBCK1 gene expression in endocardium. Red, circRNA-RBCK1; Green, vWF; Blue, nucleus. (**B** and **C**) Left ventricle wall was isolated to determine GTPCH1 mRNA by quantitative PCR in **B** and BH4 contents by HPLC in **C**. N = 10 in per group. A one-way ANOVA followed by Tukey *post-hoc* tests was used to determine *P* value in **B** and **C**. Data are presented as mean ± SD. Source data are provided as a Source Data file.

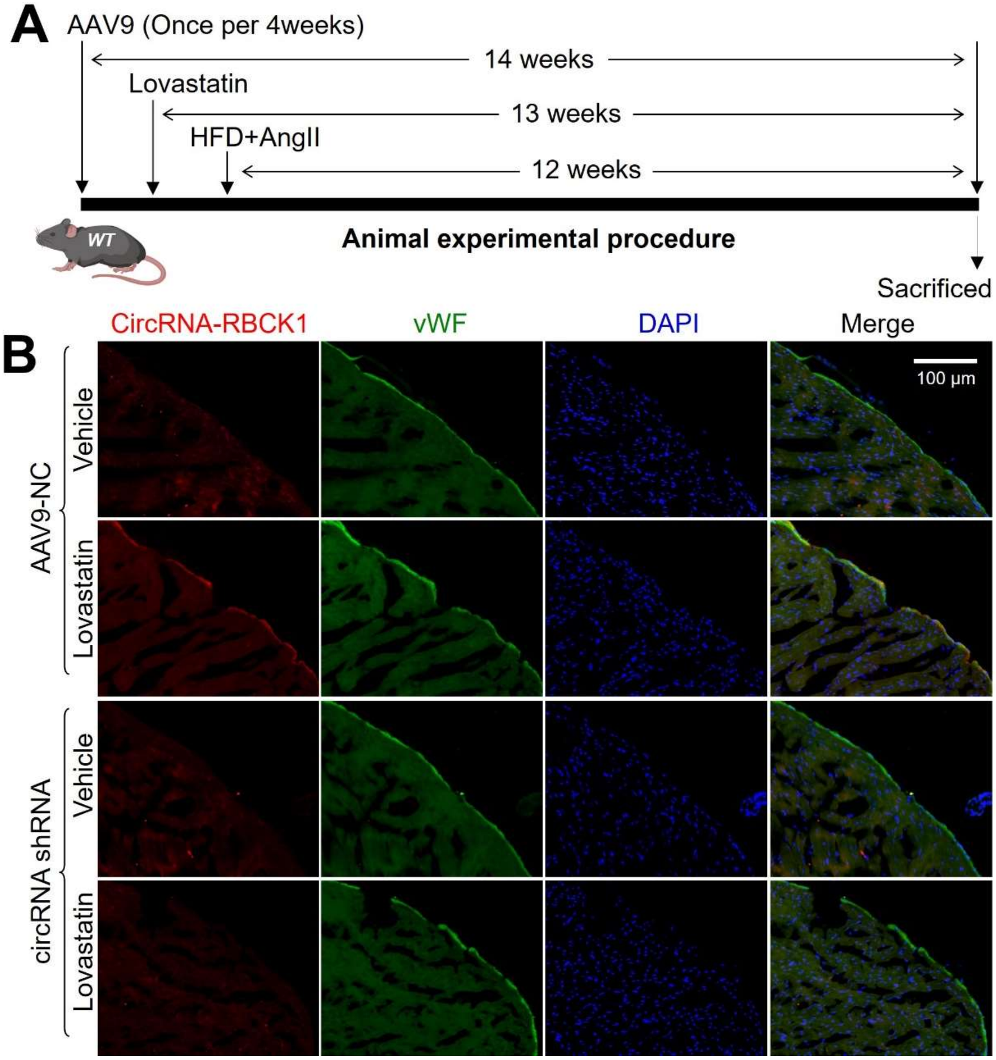

Supplementary Figure 10. AAV9-mediated gene knockdown inhibits lovastatin-induced circRNA-RBCK1 gene expression in endocardium. (**A**) Graphical procedures of animal experiments. *WT* mice were infected with adeno-associated virus 9 (AAV9) expressing negative control shRNA (AAV9-NC) or circRNA-RBCK1 shRNA (circRNA shRNA) via tail vein injection followed by 12-week HFD plus AngII with or without lovastatin administration. An injection of AAV9 was repeated once 4 weeks. Before sacrifice, conventional ECG and Doppler imaging were assessed. (**B**) Hearts isolated from mice were subjected to perform FISH analysis of circRNA-RBCK1 expression in endocardium. Red, circRNA-RBCK1; Green, vWF; Blue, nucleus. The representative pictures were shown.

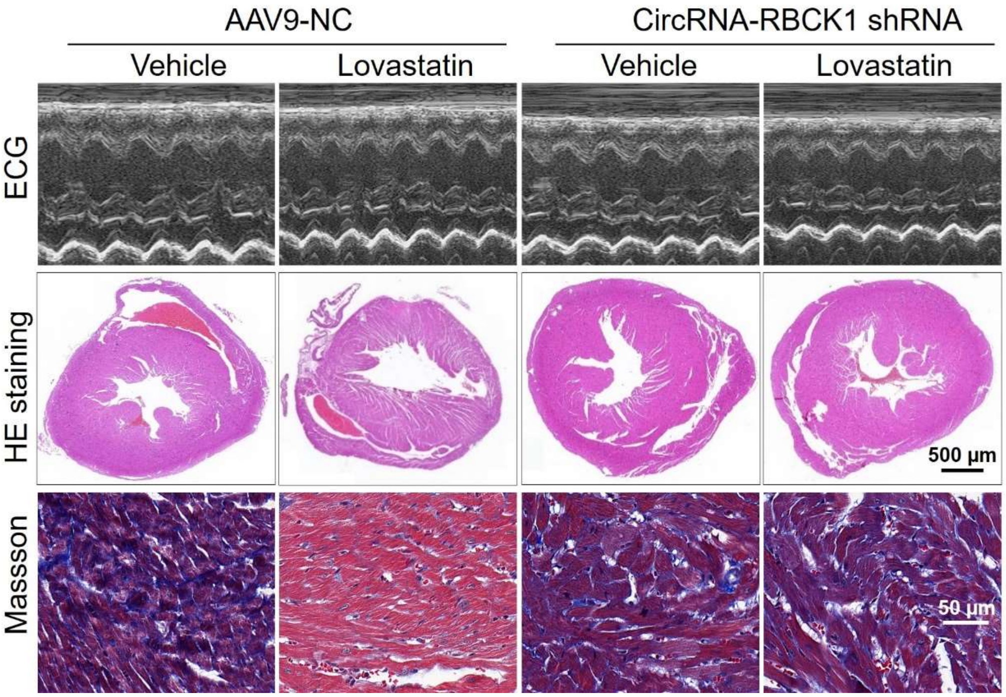

Supplementary Figure 11. AAV9-mediated gene knockdown of circRNA-RBCK1 eliminates the effects of lovastatin on cardiac hypertrophy in HFpEF mice. Graphical procedures of animal experiments were shown in Supplementary Figure 10A. Representative left ventricular M-mode echocardiographic tracings before mice were sacrificed. Hearts isolated from mice were subjected to perform HE staining and Masson staining. The representative pictures were shown. Parameters obtained from ECG were summarized in Supplementary Table 6.

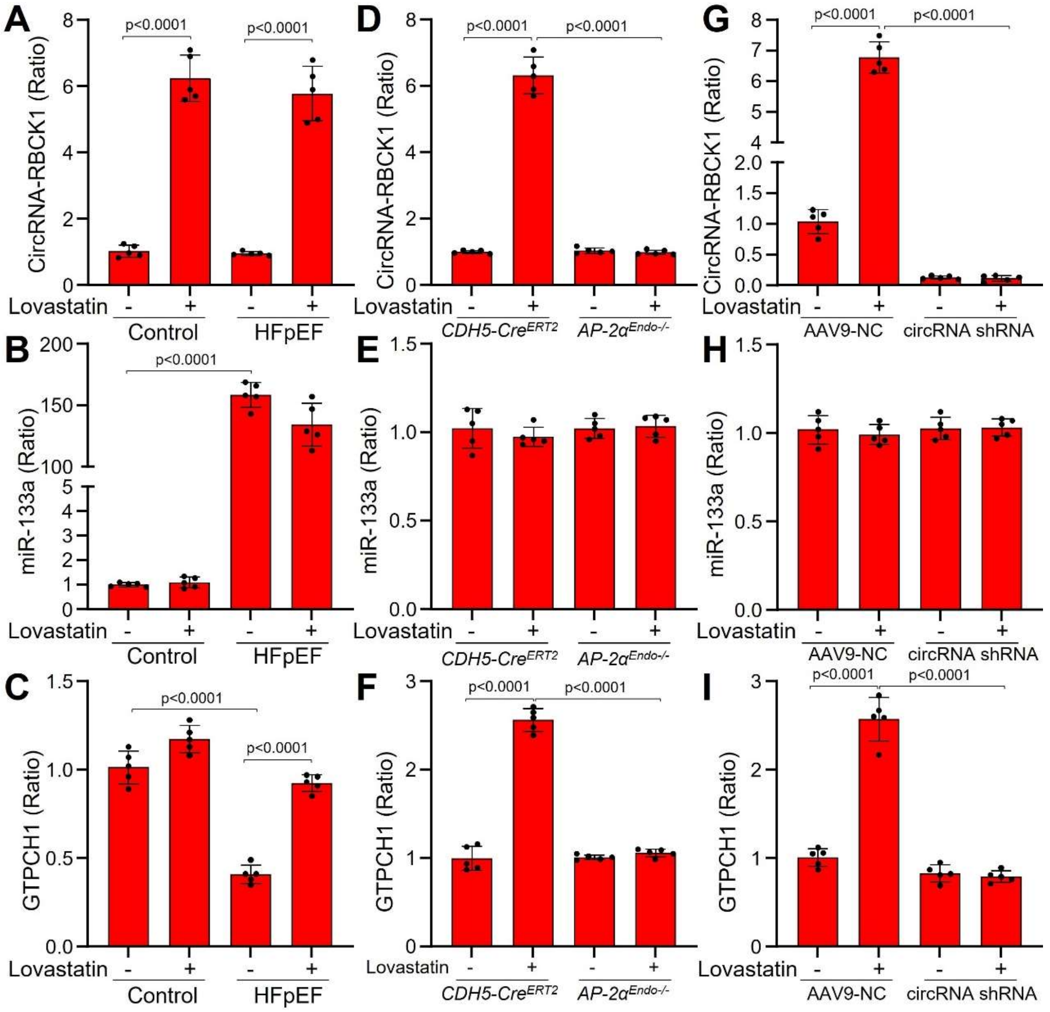

Supplementary Figure 12. Effects of lovastatin on myocardial capillary endothelial cells (MCEC) in HFpEF mice. Isolations of mouse MCECs from mice were shown in Supplementary Figure 7B. (**A-C**) Graphical procedures of animal experiments were shown in Supplementary Figure 5A. (**D-F**) Graphical procedures of animal experiments were shown in Supplementary Figure 8C. (**G-I**) Graphical procedures of animal experiments were shown in Supplementary Figure 10A. The gene expressions of circRNA-RBCK1, miR-133a, and GTPCH1 mRNA in MCEC were detected using quantitative PCR. The level in control group was setup as 1. N = 5 per group. A one-way ANOVA followed by Tukey *post-hoc* tests was used to determine *P* value in this figure. Data are presented as mean ± SD. Source data are provided as a Source Data file.

Supplementary Table 1. The effects of lovastatin on blood pressures and blood metabolism indices in mice treated with HFD plus AngII for 12 weeks

Groups	Control		HFpEF	
	**Vehicle**	**Lovastatin**	**Vehicle**	**Lovastatin**
N	10	12	13	13
Body weight (g)	21.3±1.7	19.3±1.7	31.9±2.6	29.8±1.9
Systolic blood pressure (mmHg)	103±9	98±8	152±11[a]	157±12
Diastolic blood pressure (mmHg)	72±6	67±7	94±10[b]	91±9
Fasting glucose (mM)	5.1±0.4	5.2±0.3	5.6±0.4	5.4±0.5
Cholesterol (mg/L)	508±54	575±47	981±51[c]	593±46[f]
Total triglyceride (mg/L)	681±89	609±62	983±82[d]	817±83
Low density lipoprotein (mg/L)	124±23	121±31	249±29[e]	133±37[g]
High density lipoprotein (mg/L)	267±59	229±67	286±47	275±57

The protocols and experimental designs were described in Supplementary Figure 5A. Data are expressed as means ± SD. [a]P = 0.0037, [b]P = 0.0024, [c]P = 0.0012, [d]P = 0.0056, and [e]P = 0.0062 *vs.* vehicle alone. [f]P = 0.0052 and [g]P = 0.0041 *vs.* HFpEF plus vehicle. A one-way ANOVA followed by Scheffe test was used to determine P value in this table.

Supplementary Table 2. The effects of lovastatin on echocardiographic parameters in mice treated with HFD plus AngII for 12 weeks

Groups	Control		HFpEF	
	**Vehicle**	**Lovastatin**	**Vehicle**	**Lovastatin**
N	10	12	13	13
Heart rate (bpm)	471±38	480±48	447±36	459±46
LVID,d (mm)	2.8±0.2	2.7±0.2	2.9±0.3	2.8±0.3
LVID,s (mm)	1.2±0.1	1.1±0.1	1.3±0.2	1.2±0.1
IVS,d (mm)	1.1±0.1	1.3±0.1	1.2±0.1	1.3±0.1
LVPW,d (mm)	0.9±0.1	1.1±0.1	1.0±0.1	1.2±0.1
LVFS (%)	54.7±5.5	58.6±5.9	50.7±5.1	59.1±5.7
LVEF (%)	78.6±7.1	82.1±7.8	81.3±7.8	80.2±9.1
IVRT (mm/s)	21.4±2.5	20.1±2.3	12.7±1.9[a]	21.6±2.9[d]
Peak mitral E velocity (mm/s)	434.8±57.8	424.8±37.8	986.5±78.4[b]	461.8±67.1[e]
Peak mitral A velocity (mm/s)	427.9±31.5	409.2±31.8	359.7±31.9	401.5±36.4
Peak mitral E' velocity (mm/s)	19.5±3.2	20.9±2.6	17.5±2.7	19.5±3.1
Mitral DT (ms)	22.4±2.2	23.7±2.5	14.8±1.2[c]	24.1±2.9[f]

The protocols and experimental designs were described in Supplementary Figure 5A. Data are expressed as means ± SD. [a]*P* = 0.0058, [b]*P* = 0.0071, and [c]*P* = 0.0026 *vs.* vehicle alone. [d]*P* = 0.0027, [e]*P* = 0.0016, and [f]*P* = 0.0014 *vs.* HFpEF plus vehicle. A one-way ANOVA followed by Scheffe test was used to determine *P* value in this table.

Supplementary Table 3. Blood pressures and blood metabolism indexes in tamoxifen-injected mice treated with vehicle or lovastatin under HFpEF

Groups	*CDH5-Cre^{ERT2}*		*AP-2α$^{flox/flox}$/CDH5-Cre^{ERT2}*	
	**Control**	**Lovastatin**	**Control**	**Lovastatin**
N	10	10	10	10
Body weight (g)	27.3±1.5	28.9±1.7	26.3±1.9	28.8±2.0
Systolic blood pressure (mmHg)	154±15	149±13	156±15	160±17
Diastolic blood pressure (mmHg)	97±12	97±11	99±11	95±9
Fasting glucose (mM)	5.8±0.9	5.7±0.5	5.3±0.7	5.2±0.6
Cholesterol (mg/L)	978±69	524±47[a]	955±37	515±62[c]
Total triglyceride (mg/L)	851±57	799±90	843±72	782±77
Low density lipoprotein (mg/L)	267±28	129±19[b]	271±17	135±14[d]
High density lipoprotein (mg/L)	276±31	295±27	254±30	284±30

The protocols and experimental designs were described in Supplementary Figure 8C. Data are expressed as means ± SD. [a]*P* = 0.0069 and [b]*P* = 0.0053 *vs. CDH5-Cre^{ERT2}* alone. [c]*P* = 0.0016 and [d]*P* = 0.0014 *vs. AP-2α$^{flox/flox}$/CDH5-Cre^{ERT2}* alone. A one-way ANOVA followed by Tukey *post-hoc* tests was used to determine *P* value in this table.

Supplementary Table 4. Echocardiographic and invasive haemodynamic parameters in tamoxifen-injected mice treated with vehicle or lovastatin under HFpEF

Groups	*CDH5-Cre^ERT2^*		*AP-2α^flox/flox^/CDH5-Cre^ERT2^*	
	**Control**	**Lovastatin**	**Control**	**Lovastatin**
N	10	10	10	10
Heart rate (bpm)	474±38	434±35	489±47	486±27
LVID,d (mm)	2.8±0.3	2.7±0.2	2.6±0.3	2.5±0.2
LVID,s (mm)	1.3±0.2	1.2±0.1	1.0±0.2	1.1±0.1
IVS,d (mm)	1.0±0.1	1.2±0.1	1.1±0.2	1.2±0.1
LVPW,d (mm)	1.1±0.2	1.0±0.2	1.0±0.1	1.1±0.1
LVFS (%)	54.7±6.6	55.7±5.1	57.7±5.4	53.8±5.7
LVEF (%)	81.3±7.5	79.6±7.2	85.4±7.4	83.0±7.9
IVRT (mm/s)	14.7±1.6	19.9±2.6[a]	13.6±1.2	15.8±1.6[d]
Peak mitral E velocity (mm/s)	946.1±67.7	499.8±60.1[b]	988.5±83.4	967.2±92.1[e]
Peak mitral A velocity (mm/s)	365.9±39.2	404.7±36.5	374.1±45.5	358.2±50.9
Peak mitral E' velocity (mm/s)	21.7±2.5	22.8±2.3	23.5±2.1	28.9±2.6
Mitral DT (ms)	18.8±1.7	25.9±3.2[c]	13.7±1.6	15.6±1.4[f]

The protocols and experimental designs were described in Supplementary Figure 8C. Data are expressed as means ± SD. [a]*P* = 0.0092, [b]*P* = 0.0084, and [c]*P* = 0.0066 *vs. CDH5-Cre^ERT2^* alone. [d]*P* = 0.0081, [e]*P* = 0.0017, and [f]*P* = 0.0034 *vs. CDH5-Cre^ERT2^* plus Lovastatin. A one-way ANOVA followed by Tukey *post-hoc* tests was used to determine *P* value in this table.

Supplementary Table 5. Blood pressures and blood metabolism indexes in AAV9-infected HFpEF mice treated with lovastatin

Groups	AAV9-NC		CircRNA-RBCK1 shRNA	
	**Vehicle**	**Lovastatin**	**Vehicle**	**Lovastatin**
N	11	12	13	12
Body weight (g)	29.3±2.7	30.9±2.6	29.3±2.2	32.8±2.6
Systolic blood pressure (mmHg)	148±12	152±12	155±14	157±13
Diastolic blood pressure (mmHg)	102±8	98±10	100±11	104±8
Fasting glucose (mM)	5.3±0.6	5.8±0.3	5.2±0.5	5.4±0.7
Cholesterol (mg/L)	957±54	608±51[a]	985±47	653±46[c]
Total triglyceride (mg/L)	887±89	792±82	861±72	787±80
Low density lipoprotein (mg/L)	274±13	149±24[b]	269±23	58±15[d]
High density lipoprotein (mg/L)	261±31	287±34	276±30	280±39

The protocols and experimental designs were described in Supplementary Figure 10A. Data are expressed as means ± SD. [a]*P* = 0.0073 and [b]*P* = 0.0034 *vs.* AAV9-NC plus vehicle. [c]*P* = 0.0062 and [d]*P* = 0.0053 *vs.* CircRNA-RBCK1 shRNA plus vehicle. A one-way ANOVA followed by Scheffe test was used to determine *P* value in this table.

Supplementary Table 6. Echocardiographic and invasive haemodynamic parameters in AAV9-infected HFpEF mice treated with lovastatin

Groups	AAV9-NC		CircRNA-RBCK1 shRNA	
	Vehicle	Lovastatin	Vehicle	Lovastatin
N	11	12	13	12
Heart rate (bpm)	497±59	436±47	429±67	437±57
LVID,d (mm)	2.8±0.3	2.7±0.3	2.9±0.2	2.7±0.2
LVID,s (mm)	1.2±0.3	1.1±0.2	1.2±0.2	1.3±0.1
IVS,d (mm)	1.2±0.1	1.1±0.2	1.2±0.2	1.3±0.1
LVPW,d (mm)	1.0±0.1	1.1±0.1	1.1±0.1	1.0±0.1
LVFS (%)	56.9±6.2	55.7±5.6	58.2±5.1	57.4±5.6
LVEF (%)	82.3±7.7	79.7±7.2	78.2±7.4	81.5±7.1
IVRT (mm/s)	13.5±1.4	20.8±2.1[a]	12.5±1.3	14.6±1.7[d]
Peak mitral E velocity (mm/s)	946.5±61.9	497.8±67.5[b]	989.7±84.2	951.9±90.3[e]
Peak mitral A velocity (mm/s)	331.9±30.9	417.5±32.6	379.0±38.6	357.2±49.1
Peak mitral E' velocity (mm/s)	20.4±2.2	23.6±2.7	21.3±2.1	20.5±2.6
Mitral DT (ms)	15.5±1.3	24.7±2.3[c]	13.4±1.5	14.7±1.9[f]

The protocols and experimental designs were described in Supplementary Figure 10A. Data are expressed as means ± SD. [a]$P = 0.0061$, [b]$P = 0.0045$, and [c]$P = 0.0036$ *vs.* AAV9-NC plus vehicle. [d]$P = 0.0052$, [e]$P = 0.0037$, and [f]$P = 0.0025$ *vs.* CircRNA-RBCK1 shRNA plus vehicle. A one-way ANOVA followed by Scheffe test was used to determine *P* value in this table.

Supplementary Table 7. Primers used in this study

Primer	Sequences
Human circRNA_102979	5′-ACAGGATATCGCATTGTTGG-3′<br>5′-TATACCACATGCAATTCAG-3′
Mouse circRNA_102979	5′-CCCAGGCATGCTCTCCCATC-3′<br>5′-CATTTGTGATATATGCCACC-3′
Human GTPCH1 mRNA	5’-GCCATGCAGTACTTCACCAA-3’<br>5’-AGGCTTCTGTGATGGC CACCG-3’
Mouse GTPCH1 mRNA	5′-GCTTCGGCAGCACATATACT-3′<br>5′-CGCTTCACGAATTTGCGTGT-3′
Human AP-2α mRNA	5′-CGGAATTCTGAAATATGCTATTAC-3′<br>5′-GGGATCCTCAAGATATTCACCT-3′
Human miR-125a	5′-TTGCCATAGCCATGAAGTGA-3′<br>5′-GTCTGCTGGGTCCTGTTGTT-3′
Human miR-133a	5’-AACTCCAGCTGGTCCTTAG-3’<br>5’-TCTTGAACCCTCATCCTGT-3’
Human miR-502	5′-GTCATTCCCTCTTTAATGGTG-3′<br>5′-CAGAACTTCCGCTCTAACATAC-3′
Human miR-637	5′-GTGCCGAAAGGAAGAC-3′<br>5′-TCTTTAGGGGTGTGCGTAGG-3′
Human miR-1301	5′-TGCACAGTGTCACGAACAGA-3′<br>5′-ACCTCGGAGAAGCTGAAACA-3′
Human GAPDH	5’-AGCTAAGAGAAGGGCGGAAC-3’<br>5’-CATCTGC AGGCTGACATTGA-3’
Mouse GAPDH	5′-GGAAAGCTGTGGCGTGAT-3′<br>5′-AAGGTGGAAGAATGGGAGTT-3′
U6	5’-CGCGACAAGGCCAAGAT-3’<br>5’-GCTGCTCCACCTTCTTCTG-3’
