# Supplementary material for: Statins improve cardiac endothelial function to prevent heart failure with preserved ejection fraction through upregulating circRNA-RBCK1
Source: Nat Commun. 2024 Apr 5;15:2953. doi: 10.1038/s41467-024-47327-z (PMC10997751; doi:10.1038/s41467-024-47327-z)
Supplement: Supplementary file 2 — Description of Additional Supplementary Files [file 41467_2024_47327_MOESM2_ESM.pdf]

Description of Additional Supplementary Files

File Name: Supplementary Data 1

Description: CircRNA Expression Profiling Data in HUVECs

File Name: Supplementary Data 2

Description: Transcriptional Factor Bioinformation of RBCK1 gene
